# Who gets included? Equity in digital and decentralised mental health and neurodevelopmental trials: A systematic review

**DOI:** 10.1371/journal.pdig.0001466

**Published:** 2026-06-08

**Authors:** Sophie S. Hall, Charlotte L. Hall, Christopher Partlett, Alexia Jeayes, Nikita Rattu, Helen Henshaw, Jennifer Martin, Emily Shoesmith

**Affiliations:** 1 Nottingham Clinical Trials Unit, School of Medicine, University of Nottingham, Nottingham, United Kingdom; 2 National Institute of Health and Care Research MindTech HealthTech Research Centre, School of Medicine, University of Nottingham, Nottingham, United Kingdom; 3 National Institute for Health and Care Research Nottingham Biomedical Research Centre, Institute of Mental Health, University of Nottingham, Nottingham, United Kingdom; 4 Mental Health and Clinical Neurosciences, School of Medicine, University of Nottingham, Queen’s Medical Centre, Nottingham, United Kingdom; 5 Nottinghamshire Healthcare NHS Foundation Trust, Nottingham, United Kingdom; 6 NIHR Nottingham, Nottingham Biomedical Research Centre, Ropewalk House, Nottingham, United Kingdom; 7 Hearing Science, Mental Health and Clinical Neurosciences, School of Medicine, University of Nottingham, United Kingdom; 8 Department of Health Sciences, University of York, York, United Kingdom; Schulich School of Medicine and Dentistry: Western University Schulich School of Medicine & Dentistry, CANADA

## Abstract

Decentralised clinical trials (DCTs) may help address underrepresentation in digital mental health research, but their effectiveness in reaching underserved populations is unclear. This review assessed the reporting of equity-relevant demographic data in DCTs to identify groups at risk of exclusion and barriers and facilitators to inclusive participation. A systematic search was conducted in MEDLINE, PsycINFO, Embase, CINAHL, Cochrane Central Register of Controlled Trials, and Web of Science. We included studies reporting on mental health interventions evaluated via remote, online, virtual, or hybrid DCTs, published in English from 2020-2026 (last search date: 01/07/2025), that reported participant demographics. Demographic data were extracted and summarised according to the PROGRESS-Plus framework. Demographic frequencies were compared to national population statistics. Thematic analysis identified barriers and enablers to inclusive participation in DCTs. Fifty-nine papers reporting 57 DCTs were included. Studies involved a range of mental health and neurodevelopmental conditions across the ages. Gender (100%) and age (100%) were universally reported. Reporting of other PROGRESS-Plus variables across the 57 DCTs was limited: social capital (43.9%); race/ethnicity (40.4%); occupation (36.8%); socioeconomic status (35.1%); place of residence (12.3%); religion (5.3%), and non-mental health disability (1.8%). Participants from ethnic minority backgrounds, males, unemployed individuals, and those with lower educational attainment were consistently underrepresented. While rural populations were better represented in Australian studies, data on poverty, religion, and social capital were limited and varied in representativeness. Most studies focused on adults aged 18–50 years. Thematic analysis identified key barriers including, digital exclusion, low digital literacy, cognitive and sensory challenges. Facilitators included therapist or navigator support and simplified onboarding. Equity variables are persistently underreported. DCTs do not effectively engage underserved populations in mental health research, meaning digital interventions are evaluated on unrepresentative samples. This risks perpetuating, and exacerbating, existing health inequalities, limiting the real-world impact of digital mental health solutions.

## Author summary

Decentralised clinical trials (DCTs), where research processes happen remotely, often online rather than in clinics, are promoted as a way to widen access to mental health research. We reviewed 57 DCTs focused on mental health and neurodevelopmental conditions to understand who is included in these studies and how well key demographic information is reported. Although age and gender were consistently documented, other important factors, such as ethnicity, socioeconomic status, education, and disability, were frequently missing and represented a source of exclusion. Many trials required reliable internet, specific devices, or digital skills, which may exclude people most affected by digital disadvantage. While DCTs have potential to improve inclusion, more consistent reporting and better consideration of equality, diversity and inclusion (EDI) characteristics, including digital access are needed to ensure these trials truly reach diverse populations.

## Introduction

Remote and digitised delivery of mental health care is increasingly recognised as essential to meet rising demand and improve accessibility nationally and globally [1–4]. Digital tools, such as apps, teletherapy, and AI-driven diagnostics, offer scalable, cost-effective interventions that can reduce waiting times, ease pressure on services and support personalised care [5,6]. However, for the benefits of mental health digitisation to be realised by those most at need they need be evaluated in underserved populations.

Randomised Controlled Trials (RCTs) are regarded as the ‘gold standard’ for evaluating healthcare interventions [7], but their reliance on selective samples and inadequate demographic reporting [8–10],limits real-world applicability and produces a skewed data base on which decisions are made [11–14]. Decentralised clinical trials (DCTs), also called ‘remote’, ‘online’, ‘site-less’, or ‘hybrid’ trial, conduct some or all activities outside traditional trial sites, and grew in popularity during COVID-19 [15,16]. For clarity, we use the term “decentralised clinical trials (DCTs)” throughout this review, using “remote”, “virtual”, or “online” only when referring to specific trial components such as recruitment, assessment, or intervention delivery. DCTs rely on digital technologies, offering a flexible, scalable model for evaluating digital mental health interventions, with potential to improve access, reduce costs, and support sustainability [17,18]. Whilst DCTs are increasingly positioned to promote inclusive research, evidence of their impact on equity remains uncertain, particularly in mental health, where challenges such as stigma, digital exclusion, and lack of face-to-face interaction/support can limit participation [19,20]. Neurodevelopmental conditions, such as autism and attention-deficit hyperactivity disorder (ADHD), frequently co-occur with mental health conditions [21,22] and present similar challenges [23]. In addition, requirements for digital literacy and access may further exclude disadvantaged groups disproportionately affected by mental health disorders [24–28]. Frameworks like NIHR INCLUDE and recommendations by Aiyegbusi et al. [29,30] provide guidance on engaging underserved groups, but their application to mental health DCTs is limited. Realising the inclusive potential of DCTs requires evaluating their effectiveness in reaching diverse populations and adapting designs to overcome intersecting barriers. The aims of this review were to:

1. Assess the extent to which equity-relevant data are reported in DCTs, using the Place, Race, Occupation, Gender/sex, Religion, Education, Socioeconomic status, Social capital + personas characteristics associated with discrimination (PROGRESS-plus) framework.
2. Assess representativeness as an indicator of equity by comparing PROGRESS-Plus groups with national population statistics.
3. Identify barriers and enablers to inclusive participation in DCTs in a mental health/neurodevelopmental context.

In this review, we examine equity by assessing the representativeness of participants and the completeness of PROGRESS-Plus demographic reporting.

## Materials and methods

We report methodology in accordance with the Preferred Reporting Items for Systematic Reviews (PRISMA) and Meta-analyses guidelines using the Equity extension [31] (see S1 PRISMA EQUITY CHECKLIST), following a preregistered International Prospective Register of Systematic Reviews protocol (CRD420251080321).

### Ethics statement

Not applicable for this review study

### Patient and Public Involvement (PPI)

Four PPI members with mental health and neurodevelopmental conditions (including one Black female and one Asian female) were involved in the conception of the study. Their concerns regarding inclusivity in DCT informed the development of the data extraction tool. A PPI member also reviewed the data codes derived from the thematic analysis.

### Inclusion criteria

Studies were assessed for inclusion based on the population, intervention, comparator, outcome and study design (Table 1). All included studies were confirmed to meet the predefined inclusion criteria following independent full-text review by multiple reviewers, with inclusion agreed through consensus.

**Table 1:** Inclusion criteria based on population, intervention, comparator, outcome and study design.

Population	Children (aged up to 17 years) and/or adults (aged 18 years and above) with a diagnosis of a mental health or neurodevelopmental condition (as defined by the DSM-5 [32]), in any setting.
Intervention	Studies that described or evaluated decentralised, virtual, or remote interventions (fully or partially remote), e.g., teletherapy, app-based interventions, remote assessments. Interventions must involve assessment, treatment, prevention, or management of mental health or neurodevelopmental conditions.
Comparator	Studies with the following controls were considered: normal practice (‘usual care’), waiting-list control, or any other intervention described by the authors as a comparator.
Outcomes	Studies that reported psychological, emotional and/or behavioural outcomes (e.g., agitation, anxiety, social behaviour).
Study designs	Randomised controlled trials (including randomised feasibility and pilot trials).

### Exclusion criteria

Studies were excluded if they: (a) lacked demographic data or reflections on representation; (b) involved interventions unrelated to mental health or neurodevelopmental conditions; (c) delivered non-digital or non-remote interventions; (d) had no other online trial components beyond the intervention; or (e) were published before 2020, as the COVID-19 pandemic marked a shift in DCT adoption ensuring the review reflects contemporary practices and technologies. A list of studies excluded following full-text screening, with reasons for exclusion, is provided in S3 Table. All included studies were published in peer-reviewed journals; no unpublished studies or grey literature were included in this review.

### Search strategy

**Fig 1:**
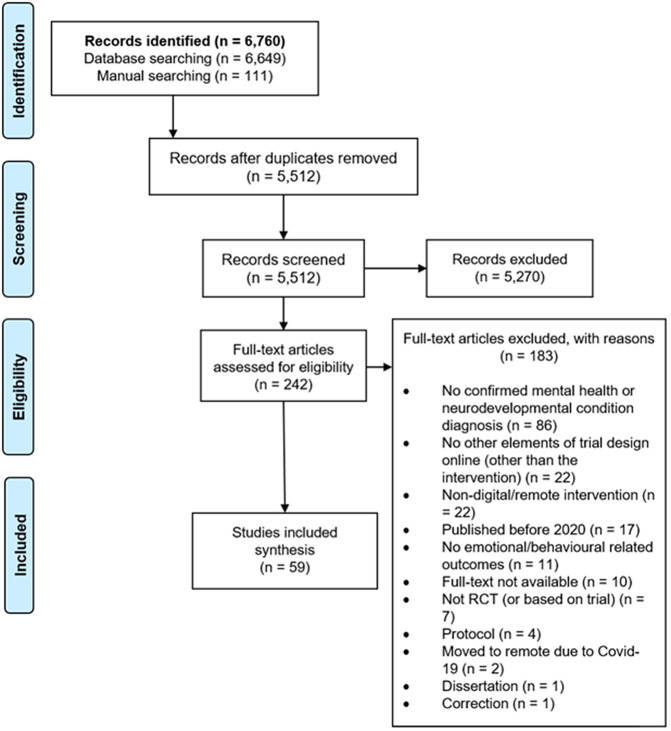
PRISMA diagram of paper selection process.

MEDLINE, PsycINFO, Embase, CINAHL, Cochrane Central, and Web of Science were searched up to 1 July 2025 using terms related to DCTs (e.g., decentralised, remote, digital), demographics (e.g., ethnicity, education), inclusivity, and mental health or neurodevelopmental conditions in children and adults. Searches were limited to English-language studies. The MEDLINE strategy is provided in S1 Table and adapted for other databases. Key journals, reference lists of included studies, and relevant systematic reviews were hand-searched. Publications were managed in Covidence (Fig 1). Titles and abstracts were independently screened by three authors (S.S.H., C.L.H., E.S.); disagreements led to inclusion in full-text review. Full texts were independently reviewed by the same authors. Inter-rater reliability during title and abstract screening demonstrated moderate agreement between reviewers, with Cohen’s kappa values ranging from κ = 0.40 to κ = 0.70, and a mean κ of 0.55. For full-text screening, agreement ranged from κ = 0.11 to κ = 0.81, with a mean κ of 0.48, indicating overall moderate agreement, although variability between reviewer pairs was observed. Discrepancies at both screening stages were resolved through discussion between reviewers to reach consensus.

### Data extraction

Using a pre-defined data extraction spreadsheet in Microsoft Excel, relevant data were extracted by four authors (S.S.H., E.S., N.R., A.J.). The data extraction fields were based on the PROGRESS-Plus equity framework [33] and aligned with systematic-review data-extraction guidance provided by the Joanna Briggs Institute (JBI) [34]. Information included research methodology, sample size, type of intervention, remote trial design elements (e.g., recruitment strategies, screening, assessments), intervention setting, digital literacy and/or access, and demographic information (e.g., race/ethnicity, gender, age, religion, occupational level, educational level) as well as any identified barriers/enablers to engagement in the trial. A complete list of data extracted is provided in S2 Table. Data extraction commenced on 14^th^ July 2025 and ended 31^st^ July 2025. No additional data were sought from study authors; all analyses were based solely on information reported in the published articles and their supplementary materials, in line with the aim of assessing reporting quality. Missing data were not imputed or supplemented; the absence of reported information was treated as an outcome reflecting reporting completeness, and no attempts were made to obtain additional data.

### Risk of bias assessment

Given the objective of this review was to evaluate representation and the quality of demographic reporting, rather than the effectiveness or validity of study findings, a formal risk of bias assessment was not undertaken. Although methodological quality tools were considered, they were not suitable in this case, since they focus on risks related to treatment effects rather than risks related to demographic completeness or representational equity. The purpose of the review was to evaluate representation, equity-related reporting, and demographic completeness, not the effectiveness or internal validity of interventions. Consistent with other equity-focused reviews [10,35],we prioritised methodological transparency in demographic reporting using PROGRESS-Plus [33], which was selected a priori and is conceptually distinct from bias appraisal of clinical effects.

### Data synthesis

We assessed reporting quality using PROGRESS-Plus guidelines [33]. Four authors (S.S.H., E.S., N.R., A.J.) independently reviewed each paper, categorising each item as reported, not reported, or unclear. Supplementary materials were accepted if explicitly referenced. PROGRESS includes place of residence, race/ethnicity, occupation, gender/sex, religion, education, socioeconomic status (SES), and social capital. For synthesis, place of residence was coded as rural/urban; occupation as employed/unemployed; education as above/below compulsory level; social capital by relationship status; and SES as low-, middle-, or high-income using country-specific definitions. SES reporting varied (income frequency, household vs. individual, currencies), and no standard cut-offs were used; where available, numbers below the national poverty line were extracted separately. The “Plus” category captured additional characteristics, including non-mental health conditions and age.

Descriptive statistics summarised demographics. Country-level synthesis included only countries represented in ≥2 studies; multi-country studies without disaggregated data were excluded. Where possible, chi-squared tests compared sample sociodemographics with national statistics. Several national statistic items required pragmatic simplification due to limited granularity in the included studies. For example, determining what counted as high- or low-income earner, we have therefore interpreted the resulting analyses cautiously and restricted conclusions to directionality (i.e., over- or under-representation), rather than precise estimates of representativeness. Perceived or measured barriers/enablers to participation were thematically analysed [36]. One author (S.S.H.) generated initial codes, which were grouped into themes. A second author (E.S.) and a PPI member independently reviewed and validated theme construction.

## Results

### Description of studies

Database and manual searching yielded a total of 6,760 records. After removal of duplicates and screening of titles, abstracts and full-text papers, 59 papers were included in the review (Fig 1), reporting a total of 57 DCTs. Two papers referred to the same DCT [37,38], but assessed different outcomes. Both DCTs met the inclusion criteria but as the data was derived from the same sample, demographics are only included once. One paper was a perspective piece containing information on relevant to inclusive participation in mental health DCTs [39]. A list of all included papers is provided in S1 Data.

Across the 57 included DCTs, sample sizes ranged from 8 to 1,225 participants (as reported at baseline). Most DCTs were conducted in the USA (n = 15; 26.3%), followed by the UK (n = 8; 14.0%), Australia (n = 7; 12.3%), Sweden (n = 4; 7.0%), and China (n = 4; 7.0%). Two DCTs (3.5%) were conducted in multiple geographic regions (e.g., USA and UK [40] and Netherlands and UK [41]. Interventions were delivered to a variety of study populations, primarily anxiety (n = 10; 17.5%), depression (n = 8; 14.0%), attention-deficit hyperactivity disorder (ADHD; n = 7, 12.3%), and autism (n = 6; 10.5%). Fourteen studies (24.6%) delivered interventions to participants with mixed diagnoses (e.g., anxiety and depression, post-traumatic stress disorder and depression, all mental health conditions).

The DCTs were split in terms of models of delivery, with 28 (49.1%) being fully remote, and 29 (50.9%) adopting a hybrid model that combined remote and in-person trial elements. Remote recruitment (e.g., via social media, online platforms, electronic health records) was reported in 40 studies (70.2%). Remote screening or eligibility assessments (e.g., via telephone, online self-reporting) were used in 40 studies (70.2%). Remote outcome assessments were performed in most studies (n = 52, 91.2%). Table 2 provides a full overview of study characteristics.

**Table 2:** Study characteristics.

Reference number	Author/Year	Country	Participants (n)	Diagnosis	Full or hybrid remote	Remote recruitment strategies (Y/N)	Remote screening and/or eligibility (Y/N)	Remote assessments/ follow-up (Y/N)
[44]	Andrews et al. (2023)	Australia	103	Anxiety or depression	Full	Yes	Yes	Yes
[75]	Backman et al. (2024)	Sweden	141	Autism	Full	Yes	Yes	Yes
[83]	Bennett et al. (2021)	UK	34	Any mental health condition	Hybrid	No	No	Yes
[95]	Bilan et al. (2025)	Spain	49	ADHD	Hybrid	No	Yes	No
[40]	Carl et al. (2020)	USA and UK	256	Generalised anxiety disorder	Full	Yes	Yes	Yes
[93]	Chan et al. (2023)	Hong Kong	320	Comorbid depressive disorder and insomnia	Full	Yes	Yes	Yes
[94]	Chien et al. (2024)	Hong Kong	50	Any neurodevelopmental conditions	Hybrid	Not clear	Not clear	Not clear
[66]	Clark et al. (2023)	UK	102	Social anxiety	Hybrid	No	No	Yes
[62]	Creswell et al. (2024)	UK	443	Anxiety	Hybrid	No	Yes	Yes
[91]	Dopfner et al. (2025)	Germany	431	ADHD	Hybrid	No	Yes	Yes
[77]	Eto et al. (2025)	Japan	30	ADHD	Full	Yes	Yes	Yes
[41]	Eylem et al. (2021)	Netherlands and UK	18	Suicidal ideation	Hybrid	Yes	Not clear	Yes
[78]	Fatouros et al. (2025)	Greece	200	Depression and generalised anxiety disorder	Full	Yes	Yes	Yes
[37,38]	Felder et al. (2020, 2022)	USA	208	Insomnia	Full	Yes	Yes	Yes
[69]	Grenier-Martin et al. (2022)	Canada	29	Intellectual and developmental disability	Hybrid	Yes	Not clear	Yes
[52]	Guzick et al. (2023)	USA	8	ADHD	Hybrid	Not clear	Yes	Yes
[60]	Guzick et al. (2024)	USA	57	ADHD	Full	Yes	Yes	Yes
[39]	Hall et al. (2024)	N/A	N/A	Across all conditions	N/A	N/A	N/A	N/A
[86]	Hartley et al. (2022)	Australia	16	Autism	Hybrid	Yes	Not clear	Yes
[42]	Haun et al. (2023)	USA	265 dyads	PTSD	Full	Yes	Yes	Yes
[43]	He et al. (2022)	China	148	Depression	Hybrid	Yes	Not clear	Yes
[49]	Heller et al. (2020)	Netherlands	159	Depression and anxiety	Hybrid	Yes	Not clear	Yes
[72]	Hoffmann et al. (2021)	Denmark	101	Anxiety	Full	Yes	Yes	Yes
[63]	Hollis et al. (2021)	UK	224	Tourette’s syndrome	Hybrid	Yes	No	Yes
[53]	Huberty et al. (2021)	USA	239	Insomnia	Full	Yes	Yes	Yes
[80]	Jamali et al. (2022)	Iran	43	Autism	Full	Yes	Yes	Yes
[51]	Jent et al. (2021)	Spain	130	Disruptive behaviour	Hybrid	Not clear	Yes	Yes
[54]	Kalmbach et al. (2020)	USA	91	Insomnia	Full	Yes	Yes	Yes
[84]	Kandola et al. (2024)	UK	908	Depression	Full	Yes	Yes	Yes
[61]	Kenworthy et al. (2023)	USA	97	Autism	Hybrid	Not clear	No	Yes
[79]	Kwon et al. (2024)	Korea	74	ADHD	Hybrid	No	No	Yes
[48]	Lewis et al. (2024)	Australia	61	Autism	Full	Yes	Yes	Yes
[81]	Lindgren et al. (2020)	USA	38	Autism	Hybrid	Not clear	Not clear	Yes
[70]	Lippke et al. (2021)	Germany	300	Psychosomatic diagnosis	Hybrid	No	No	Yes
[55]	Malarkey et al. (2024)	USA	125	Insomnia	Hybrid	Yes	Not clear	Yes
[46]	March et al. (2023)	Australia	137	Anxiety	Hybrid	Not clear	Yes	Yes
[45]	March et al. (2025)	Australia	137	Anxiety	Full	Yes	Yes	Yes
[85]	McCloud et al. (2020)	UK	168	Anxiety and depression	Full	Yes	Yes	Yes
[47]	McLellan et al. (2024)	Australia	95	Anxiety	Hybrid	No	Yes	Yes
[88]	Mechler et al. (2022)	Sweden	272	Depression	Full	Yes	Yes	Yes
[92]	Moshe et al. (2022)	Germany	253	Depression	Hybrid	Yes	Not clear	Yes
[87]	Murray et al. (2021)	Australia	302	Bipolar disorder	Full	Yes	Yes	Yes
[73]	Nardi et al. (2022)	USA	27	Generalised anxiety disorder	Hybrid	Yes	No	Yes
[76]	Nissling et al. (2023)	Sweden	52	Anxiety	Full	Yes	Yes	Yes
[74]	Nordh et al. (2021)	Sweden	103	Social anxiety disorder	Hybrid	Yes	Yes	Not clear
[56]	Ong et al. (2024)	USA	178	PTSD and/or depression	Full	Yes	Yes	Yes
[57]	Piscitello et al. (2024)	USA	43	ADHD	Hybrid	Yes	Yes	Not clear
[58]	Possemato et al. (2022)	USA	81	Depression, anxiety and PTSD	Full	Yes	Yes	Yes
[64]	Richards et al. (2020)	UK	361	Depression and anxiety	Full	Yes	Yes	Yes
[82]	Sabri et al. (2025)	USA	144	PTSD and/or depression	Full	Yes	Yes	Yes
[65]	Sayal et al. (2025)	UK	1225	Emotional difficulties	Hybrid	No	Yes	Yes
[59]	Segal et al. (2020)	USA	460	Depression	Hybrid	Yes	Yes	Not clear
[71]	Seo et al. (2022)	South Korea	73	Postpartum depression	Full	Yes	Yes	Yes
[67]	Sun et al. (2021)	China	168	Perinatal depression	Hybrid	Not clear	Yes	Yes
[50]	Tan et al. (2023)	Malaysia	48	Depression and anxiety	Hybrid	No	No	Yes
[89]	Tan et al. (2024)	China	69	Learning disabilities	Full	Yes	Yes	Yes
[68]	Wong et al. (2021)	Hong Kong	79	Depression	Full	Yes	Yes	Yes
[90]	Wu et al. (2023)	China	93	OCD	Full	Yes	Yes	Yes

### To what extent are equity-relevant data reported in DCTs in mental health, as defined by the PROGRESS-Plus guidelines?

In line with the PROGRESS-Plus guidelines, 58 of the included 59 papers (98.3%) reported participant demographics. One study did not report demographic characteristics, as this was a perspective piece, for which reporting was not applicable [39].Two papers reported on the same DCT, therefore demographics summarised below are taken from 57 DCTs [37,38]. The most frequently reported PROGRESS-Plus items related to gender (100%) and age (100%). The least frequently reported PROGRESS-Plus items related to non-mental health disabilities (1/57: 1.8%), religion (3/57: 5.3%) and place of residence or setting (rural vs. urban areas) (7/57: 12.3%) (Table 3).

**Table 3:** Number of DCTs (total n = 57) reporting PROGRESS-Plus items.

PROGRESS-Plus item	Number of studies reporting demographic information n (%)	Citations
Place (rural vs urban)	7 (12.3%)	[42–48]
Race/ethnicity	23 (40.4%)	[37,38,40,42,43,49,51–67]
Occupation	21 (36.8%)	[41,44,49,50,55,58,59,62–64,66,68–77]
Gender	57 (100%)	[37–95]
Religion	3 (5.3%)	[50,64,71]
Education	41 (71.9%)	[37,38,40–44,47–52,55,57–63,66,68–77,80,82,85,86,89–94]
Socioeconomic status	20 (35.1%)	[37,38,44–48,52,54,57,59,62,65,67–71,73,89,94]
Social capital	25 (43.9%)	[37,38,40–42,44,47,48,50,57–59,62,66–68,70,72,76,77,82,89,90,92–94]
Age	57 (100%)	[37–95]
Non mental health disability	1 (1.8%)	[71]

Demographic information from all included DCTs, disaggregated by country, is presented below. Descriptive statistics are limited to countries represented in at least two DCTs. Studies conducted across two or more countries in which data were not reported separately by country are excluded. A comprehensive overview of demographic information reported in each included DCT is available in S4 Tables (S4a) alongside documentation of the source data used to acquire the representation of each sociodemographic variable by country (S4b).

*Place:* Seven DCTs (12.3%) reported participants’ place of residence categorised as rural or urban. Among the 1,211 participants reported in these seven studies, 626 (51.7%) resided in urban areas, 306 (25.3%) in rural areas, and data for 279 participants (23.0%) were either missing or not reported. One of the seven DCTs was in the USA [42] and one in China [43] demographics for these counties are reported in S4 Table but were insufficient for further comparison. The remaining five DCTs were conducted in Australia [44–48], encompassing 533 participants: 260 (48.8%) lived in urban areas (population statistics: 86.5%), 160 (30.0%) in rural areas (population statistics: 13.5%), and residence data were unavailable for 113 participants (21.2%). Chi-squared tests identified there were significantly greater representation of rural based participants in these Australian studies compared to general population statistics (*p <* .001).

*Race/ethnicity:* Ethnicity data were reported in 23 DCTs (40.4%), encompassing a total of 5,381 participants. Among these, 4,054 individuals (75.3%) were identified as White, 493 (9.2%) as Asian, 268 (4.9%) as Black, 208 (3.9%) as ‘Other’, and 152 (2.8%) as of ‘Multiple’ ethnicities. From the 23 studies, ethnicity information was either missing or not reported for 261 participants (4.9%). Three DCTs were the only representation from that country [49–51], and one DCT reported demographics aggregated across multiple countries without separating data by country [40]. Therefore, the tabulated papers include DCTs conducted in the USA [37,42,52–61], the UK [62–66], and China [43,67] (Table 4).It was not possible to extract ethnicity of the population in China as China only officially report Han or non-Han statistics. Chi-squared test revealed a statistically significant difference between the racial representation reported in the included studies and national population statistics in the UK and USA, indicating over representation of white participants relative to their population proportions (*p’s* < .0001).

**Table 4:** Ethnicity reported across the included studies and separated by countries (represented in ≥2 DCTs).

	Total participants (n)	White n (%)	Asian n (%)	Black n (%)	Multiple n (%)	Other n (%)	Missing/not reported n (%)
Across all geographic regions (n = 23*)	5,381*	4,054 (75.3%)	493 (9.2%)	268 (4.9%)	152 (2.8%)	208 (3.9%)	261 (4.9%)
USA (n = 12)	2,117	1,564 (73.9%)	72 (3.4%)	224 (10.6%)	55 (2.6%)	129 (6.1%)	128 (6.0%)
USA Population Statistics^1^		61.6%	4.8%	12.4%	10.2%	0.1%	
UK (n = 5)	2,355	2,011 (85.4%)	60 (2.5%)	18 (0.8%)	64 (2.7%)	72 (3.1%)	130 (5.5%)
UK Population Statistics^1^		81.7%	9.3%	4%	2.9%	2.1%	
China (n = 2)	316	0 (0.0%)	305 (96.5%)	0 (0.0%)	0 (0.0%)	0 (0.0%)	11 (3.5%)

*Including four DCTs that are only represented by one country or combined countries. ^1^ Population statistics are in supplementary material (S4 Table).

*Occupation*: Of the 21 DCTs (36.2%) that reported participants’ occupational status, a total of 3,059 individuals were classified as either ‘employed’ (n = 2023, 66.1%) or ‘not employed’ (n = 649, 21.2%). From the 21 DCTs, occupational status data were missing or unreported for 387 participants (12.7%). Only one DCT, conducted in Hong Kong, provided detailed information on occupational type [68]. Nine DCTs were the only representation from that country [44,49,50,68–72], and one DCT reported demographics aggregated across multiple countries without separating data by country [41]. Therefore, the tabulated papers include DCTs conducted in the USA [55,58,59,73], the UK [62–64,66], and Sweden [74–76] (Table 5).There was a statistically significant difference between the representation of employed and unemployed participants reported in the included studies and national population statistics for all three countries (*p’s* < .001), with the data indicating underrepresentation of unemployed individuals relative to their population proportions (*p’s* < .0001).

**Table 5:** Occupational status reported across the included studies and separated by countries (represented in ≥2 DCTs).

	Total participants (n)	Employed n (%)	Not employed n (%)	Missing/not reported n (%)
Across all geographic regions (n = 21*)	3,059*	2,023 (66.1%)	649 (21.2%)	387 (12.7%)
USA (n = 4)	693	438 (63.2%)	234 (33.8%)	21 (3.0%)
USA Population Statistics^1^		52.7%	42.8%	
UK (n = 4)	1,130	857 (75.8%)	174 (15.4%)	99 (8.8%)
UK Population Statistics^1^		75.1%	24.9%	
Sweden (n = 3)	296	129 (43.6%)	64 (21.6%)	103 (34.8%)
Sweden Population Statistics		69%	31%	

*Including 10 DCTs that are only represented by one country or combined countries. ^1^ Population statistics are in supplementary material (S4 Table).

*Gender:* Among DCTs that reported gender (n = 57; 100%), a total of 10,4331 participants were included. Of these, 6,685 (64.3%) identified as female, 2,691 (25.9%) as male, 30 (0.3%) as non-binary, and 15 (0.1%) as ‘other’. Gender data were missing or not reported for 980 participants (9.4%) across the included DCTs reporting Gender. Nine DCTs were the only representation from that country [49,50,69,71,72,77–80], and two DCTs reported demographics aggregated across multiple countries without separating data by country [40,41]. Therefore, the tabulated papers include studies conducted in the USA [37,42,52–61,73,81,82], the UK [62–66,83–85], Australia [44–48,86,87], Sweden [74–76,88], China [43,67,89,90], Germany [70,91,92], Hong Kong [68,93,94], and Spain [51,95] (Table 6). Chi-squared test revealed a statistically significant difference between the representation of male and female participants reported in the included studies and national population statistics for all countries (*p’s* < .001) except Hong Kong (*p =* 0.5), which has a comparatively small sample size, with the data indicating underrepresentation of males relative to their population proportions. It should be noted that despite the small sample size, Spain was significantly different to expected population statistics in the opposite manner to the other studies, with females being underrepresented. However, this is likely due to a reflection on poor reporting as one study [51] only reported the number of males participating, contributing to a comparably large amount of ‘missing/not reported’ data, as we cannot assume the remaining sample are female.

**Table 6:** Gender and sex reported across the included studies and separated by countries (represented in ≥2 DCTs).

	Total participants (n)	Females n (%)	Males n (%)	Non-binary n (%)	Other n (%)	Missing/not reported n (%)
Across all geographic regions (n = 57*)	10,331*	6,674 (64.6%)	2,632 (25.5%)	30 (0.3%)	15 (0.1%)	980 (9.5%)
USA (n = 15)	2,326	1,542 (66.3%)	765 (32.9%)	0 (0.0%)	3 (0.1%)	16 (0.7%)
USA Population Statistics^1^		50.5%	49.5%			
UK (n = 8)	3,465	2,173 (62.7%)	700 (20.2%)	30 (0.9%)	3 (0.1%)	559 (16.1%)
UK Population Statistics^1^		49.6%	51.4%			
Australia (n = 7)	851	568 (66.7%)	271 (31.8%)	0 (0.0%)	3 (0.4%)	9 (1.1%)
Australia Population Statistics^1^		50.7%	49.3%			
Sweden (n = 4)	568	441 (77.6%)	118 (20.8%)	0 (0.0%)	0 (0.0%)	9 (1.6%)
Sweden Population Statistics^1^		49.7%	50.3%			
China (n = 4)	478	327 (68.4%)	151 (31.6%)	0 (0.0%)	0 (0.0%)	0 (0.0%)
China Population Statistics^1^		48.8%	51.2%			
Germany (n = 3)	984	740 (75.2%)	143 (14.5%)	0 (0.0%)	0 (0.0%)	101 (10.3%)
Germany Population Statistics^1^		50.6%	49.4%			
Hong Kong (n = 3)	449	136 (30.3%)	102 (22.7%)	0 (0.0%)	0 (0.0%)	211 (46.9%)
Hong Kong Population Statistics^1^		55%	45%			
Spain (n = 2)	179	6 (3.4%)	92 (51.3%)	0 (0.0%)	0 (0.0%)	81 (45.3%)
Spain Population Statistics^1^		50.9%	49.1%			

*Including 11 DCTs that are only represented by one country or combined countries. ^1^ Population statistics are in supplementary material (S4 Table).

*Religion:* Only three DCTs (5.3%), across different countries, reported data on participants’ religious affiliation, comprising a total of 482 participants. One DCTs conducted in the UK [64] included 361 participants, of whom 221 (61.2%) reported no religious affiliation, 88 (24.4%) identified as Christian, and 52 (14.4%) reported their religion as ‘other’. A DCT in South Korea [71] included 73 participants, with 38 (52.1%) reporting no religion, 18 (24.7%) identifying as Buddhist, 13 (17.8%) as Christian, and 4 (5.5%) as Catholic. Lastly, in a DCT conducted in Malaysia [50], 38 of the 48 participants (79.2%) identified as Muslim, while 10 (20.8%) reported their religion as ‘other’. There were no missing data on religious affiliation in any of the three studies that reported this information.

*Education:* Forty-one DCTs (72.0%) reported educational attainment. However, three of these presented only the mean number of years of education, which could not be categorised into educational levels [61,77,90]. Therefore, 38 DCTs were classified based on whether participants had completed only compulsory education or education beyond the compulsory level (reporting on 5,698 participants). Of these, 4,310 (75.6%) participants had completed education beyond the compulsory level, 1,185 (20.8%) participants had completed compulsory education (e.g., primary and/or secondary school). Educational data were missing or not reported for 203 (3.6%) participants across the 41 DCTs.

Seven DCTs were the only representation from that country [49–51,69,71,72,80], and two reported demographics aggregated across multiple countries without separating data by country [40,41]. Therefore, the tabulated papers include DCTs conducted in the USA [37,42,52,55,57–60,73,82], the UK [62,63,66,85], Australia [44,47,48,86], Germany [70,91,92], Hong Kong [68,93,94], Sweden [74–76], and China [43,89]. On average, across these DCTs, more than half of the participants had pursued education beyond the compulsory level. Only studies conducted in Germany and Sweden had samples in which the majority of participants had not continued their education beyond the compulsory level (Table 7). The chi-squared test indicated that participants who pursued education beyond the compulsory level were overrepresented compared to national population statistics in all countries (*p’s* < .001), except for Germany (*p =* 0.3) and Sweden (*p =* 0.9), where the difference was not statistically significant.

**Table 7:** Educational data reported across the included studies and separated by countries (represented in ≥2 DCTs).

	Total participants (n)	Compulsory n (%)	Higher n (%)	Missing/not reported n (%)
Across all geographic regions (n = 38*)	5,698	1,185 (20.8%)	4,310 (75.6%)	203 (3.6%)
USA (n = 10)	1,683	152 (9.0%)	1,461 (86.8%)	70 (4.2%)
USA Populations Statistics^1^		39%	61%	
UK (n = 4)	937	141 (15.0%)	796 (85.0%)	0 (0.0%)
UK Population Statistics^1^		66%	33.8%	
Australia (n = 4)	275	26 (9.5%)	249 (90.5%)	0 (0.0%)
Australia Population Statistics^1^		16%	84%	
Germany (n = 3)	984	602 (61.2%)	377 (38.3%)	5 (0.5%)
Germany Population Statistics^1^		63%	37%	
Hong Kong (n = 3)	449	33 (7.3%)	389 (86.6%)	27 (6.1%)
Hong Kong Population Statistics^1^		59%	41%	
Sweden (n = 3)	296	138 (46.6%)	94 (31.8%)	64 (21.6%)
Sweden Population Statistics^1^		60%	40%	
China (n = 2)	217	2 (0.9%)	215 (99.1%)	0 (0.0%)
China Population Statistics^1^		39%	61%	

*Including 9 DCTs that are only represented by one country or combined countries. ^1^ Population statistics are in supplementary material (S4 Table).

*Socioeconomic status:* Twenty DCTs reported participant income, however, one DCT reported frequency of financial stress and therefore was not included in this analysis [82], (n = 19 DCTs). There was only one DCT conducted in each the following countries: Canada, Germany, South Korea [69–71]. Five DCTs conducted in Australia [44–48] are included in Table 8, of which two reported Socio-economic Index for Areas percentiles [45,46] making classification more reliable. One DCT in China reported income in USD ($) [67] and one in RMB (Chinese Yuan) [50]. One DCT in Hong Kong reported the number of participants with a monthly family income equal to or greater than the median (classified as middle) [94] and the other reporting monthly family income in HK$ [68]. One UK DCT reported Index of Multiple Deprivation quintiles [65] and one reported annual total household income [62]. Of the five USA based DCTs, one reported the number of participants who had an income ≥ $100,000 [37], these were classified as middle SES for the purpose of the table. One DCT only reported participants who were in poverty [54], and these were classified as low SES in the table. The remaining three DCTs reported annual income [57,59,73]. The data sources used for classification of income for each country is presented in S5 Table.

**Table 8:** Socioeconomic status as calculated based on area of deprivation status or income reported across the included studies and separated by countries (represented in ≥2 DCTs).

	Total Participants (n)	Low n (%)	Middle n (%)	High n (%)	Missing/not reported n (%)
Across all geographic regions (n = 19*)	3798	1087 (28.6%)	1203 (31.7%)	999 (26.3%)	509 (13.4%)
Australia (n = 5) ^1^	533	121 (22.7%)	201 (37.7%)	178 (33.4%)	33 (6.2%)
China (n = 2) ^1^	237	84 (35.4%)	107 (45.1%)	30 (12.7%)	16 (6.7%)
Hong Kong (n = 2) ^1^	129	65 (50.4%)	35 (27.1%)	36 (12.7%)	23 (6.8%)
UK (n = 2) ^1^	1668	539 (32.3%)	330 (19.8%)	629 (37.7%)	170 (10.2%)
USA (n = 5) ^1^	829	88 (10.6%)	468 (56.4%)	124 (15%)	149 (17.8%)

*Including 3 DCTs that are only represented by one country; ^1^ Population statistics are in supplementary material (S5).

It was possible to extract a proxy for the number of participants potentially living in poverty as per their country’s definition for seven DCTs, including three in Australia [44–46], one in Hong Kong [68], two in the UK [62,65] and one in the USA [54]. This data is presented in Table 9. See S5 Table (S5b) for information on the classifications. Chi-squared tests were not computed on this sub-selection of papers. The data indicates considerable variation amongst papers in the degree to which the sample is representative of national poverty statistics.

**Table 9:** Number and % living in poverty represented in the sample.

Author	Country	n (%) in poverty
March 2025	Australia	15 (10.9%)
March 2023	Australia	12 (8.7%)
Andrews 2023	Australia	15 (14.5%)
Total	Australia	42 (11.1%)
Australia Population Statistics^1^		13.4%
Wong 2021	Hong Kong	39 (49.3%)
Hong Kong Population Statistics^1^		20.2%
Creswell 2024	UK	35 (7.9%)
Sayal 2025	UK	214 (17.4%)
Total	UK	249 (14.9%)
UK Population Statistics^1^		15%
Kalmbach	USA	16 (17.5%)
USA Population Statistics^1^		11.4%

^1^Population statistics are in supplementary material (S5).

*Social capital:* Of the 25 DCTs (43.9%) that reported on social capital, 21 categorised participants by relationship status (e.g., married or in a relationship, single, divorced, separated, or widowed), while four DCTs used living arrangements as the categorisation (e.g., living alone, with family, or with other adults). Among the 21 DCTs that reported relationship status, data were reported for 3,531 participants. Of these, 2,106 (59.6%) were married, in a civil partnership, or in a long-term relationship; 470 (13.3%) were single and/or never married; 300 (8.5%) were divorced, separated, or widowed; and 2 participants (0.1%) chose not to disclose this information. Relationship status data were missing or not reported for 653 participants (18.5%).

Three DCTs were the only representation from that country [50,72,77], and two DCTs reported demographics aggregated across multiple countries without separating data by country [40,41]. Therefore, the tabulated papers include DCTs conducted in the USA [37,42,57–59,82], China [67,89,90], Hong Kong [68,93,94], the UK [62,66], and Germany [70,92] (Table 9). National population statistics were not possible to obtain for China and Hong Kong. For the DCTs in UK, USA and Germany there was a greater representation of individuals who were married than would be expected based on their countries population statistics (*p* < .0001). See Table 10.

**Table 10:** Social capital reported across the included studies and separated by countries (represented in ≥2 DCTs).

	Total participants (n)	Married, civil partnership or relationship (n, %)	Never married or single (n, %)	Divorced, separated or widowed (n, %)	Prefer not to say (n, %)	Missing/not reported (n, %)
Across all geographic regions (n = 21*)	3,531	2,106 (59.6%)	470 (13.3%)	300 (8.5%)	2 (0.1%)	663 (18.5%)
USA (n = 6)	1,201	765 (63.7%)	175 (14.6%)	233 (19.4%)	0 (0.0%)	28 (2.3%)
USA Marriage Population Statistics^1^		49.4%				
China (n = 3)	330	285 (86.4%)	37 (11.2%)	4 (1.2%)	0 (0.0%)	4 (1.2%)
Hong Kong (n = 3)	449	89 (19.8%)	59 (13.1%)	2 (0.4%)	0 (0.0%)	299 (66.6%)
UK (n = 2)	545	408 (74.9%)	0 (0.0%)	0 (0.0%)	0 (0.0%)	137 (25.1%)
UK Marriage Population Statistics^1^		46.9%				
Germany (n = 2)	553	337 (60.9%)	34 (6.1%)	39 (7.1%)	0 (0.0%)	143 (25.9%)
Germany Marriage Population Statistics^1^		51%				

*Including 5 DCTs that are only represented by one country or combined countries; ^1^ Population statistics are in supplementary material (S4 Table).

Four DCTs reported social capital in terms of living arrangements. Three of these were conducted in Australia. One DCT [44] reported that participants lived with a mean of 1.67 other residents. Another found that 9 of 61 participants (14.8%) were from single-parent households [48], while a third reported that 86 of 95 participants (90.5%) were from two-parent households [47]. The fourth DCT, conducted in Sweden [76], reported that among 52 participants, 47 (90.4%) lived with a family member, 1 (1.9%) with another adult, 2 (3.8%) lived alone, and data were missing for 2 participants (3.8%).

*Non-mental health conditions:* Only one DCT, which was conducted in South Korea [71], reported on ‘diagnosis of physical illness (Yes: n = 9, 12.3%).

*Age:* Age was predominantly reported in terms of mean values therefore it was not possible to categorise the sample accurately into age-brackets. From the 59 papers included in the review, age was presented in 58 papers (one being a perspective piece), but one included the duplication of the same sample (n = 57 studies). The majority of the DCTs delivered interventions to adults (34/57: 59.6%), followed by children under 15 years of age (18/57: 31.6%), two DCTs conducted in Sweden tested the intervention in adolescence (15–19 years) (2/57: 3.5%) [76,88] and a third DCT in Sweden involved both younger adults and adolescence (16–25 years) (1/57: 1.8%) [75]. Two other DCTs included a mixed age population of adults and children (1/57: 1.8%) [86] and children and adolescence (1/57: 1.8%) [65].

*Digital literacy/access:* Digital access was a common inclusion criterion for the DCTs (29/57) as well as being mentioned in the perspective piece [39]. Typically participants were required to have a computer, smartphone, or tablet with stable internet [41,47,65,66,72,74]. Some DCTs specified device types (e.g., Apple/Android smartphones), specific internet speeds, or a regularly used email [55,81,85,93] and a county specific (Australian) IP address [45]. Reliable access to both internet and phone was also required in some cases [55]. Technical issues were frequently reported, including connectivity problems and device limitations, which disrupted interventions and may have contributed to dropout [77,86].

The role of digital skills was only considered by the perspective piece [39] and 7/57 trials, typically in the form of assessing digital literacy/competence through self-report or purpose-built questionnaires [42,75,85,87,92]. Some DCTs reported challenges when participants lacked digital familiarity, such as difficulties using computers [70,90], but only one study [70] incorporated support mechanisms, such as how to check internet connectivity. The recent perspective piece [39] highlighted the importance of structured support programs offering digital skills training both online and in person. It also identified digital navigators—clinic staff trained to provide technical assistance—as a promising way to support users without overburdening clinical teams. These studies highlight that digital access and literacy are critical factors for equitable access and engagement in digital mental health research and interventions.

### What are the identified barriers and enablers to participation in DCTs in a mental health context?

#### High dropout and recruitment challenges.

Across the included trials, 13 of 57 (22.8%) reported challenges in keeping participants engaged or enrolled. High dropout rates were described in 8 trials [42,43,46,69,83–85,90], and 3 trials (5.3%) reported difficulties with recruitment linked to stigma or low mental health literacy [41,49,86]. Participant dropout was associated with feeling confused or unsupported [46,83], mistaking surveys for the intervention or wanting more personal contact during onboarding [42], difficulty with digital tools [90], and individual factors such as age, education, and social support [59,92]. However, measuring engagement itself was challenging, as trials used inconsistent metrics (e.g., clicks, time spent, pages visited), limiting comparability and obscuring best-practice approaches [39].

#### Support/navigators.

Support from therapists or trained staff consistently emerged as a key facilitator of engagement in digital mental health trials. Participants often preferred therapist-assisted formats [44], and regular check-ins, whether via email or phone, helped sustain involvement [52,78]. Eto et al demonstrated that adapting face-to-face therapy techniques for digital delivery, such as co-creating worksheets and focusing on patient interests, could enhance engagement [77]. In culturally specific contexts, like Muslim communities, directive guidance was especially valued, with unclear instructions perceived as incompetence [41].

Beyond therapeutic input, practical support and project navigators played a crucial role from on-boarding, technical demonstrations, through to reminders to prevent disengagement [42,45,78,83,85]. However, in resource-limited settings or among people with severe anxiety, therapist-led models may not be feasible [39]. Additional strategies to boost engagement were suggested included motivational interviewing, short instructional videos, and text reminders [51]. However, not all digital support features, such as social networking tools, will be effective or accepted by all [86].

#### Participant burden.

The burden of participation, whether cognitive, emotional, sensory, or logistical, was another major barrier. Complex onboarding processes, long sessions, and excessive paperwork discouraged engagement [42] and participants dropped out due to tedious content or lack of engagement [43,80]. In particular, children may struggle with focus and motivation [86] and some participants found digital formats (e.g., reading on screens) uncomfortable or inaccessible [90].

To mitigate these issues, studies recommended simplifying onboarding, reducing the use of passwords, digitising forms [39,42] and simplifying data collection methods including the numbers of measures required [83]. More specific recommendations were identified in one paper, which reported that expert as opposed to caregiver/lay explanations and demonstrations supported participant engagement with the intervention and short videos (~2 minutes) were more engaging than longer content [51]. A further paper highlighted the potential of gamification and repeatable content may sustain interest, but this was not assessed directly [86]. Furthermore, online formats were beneficial to aspects of participant burden, including that associated with commuting time, reducing stigma, and supporting a more comfortable therapeutic relationship—possibly due to the “online disinhibition effect [70].

## Discussion

This review of 59 papers (reporting 57 RCTs and one perspective piece) found that, although DCTs are increasingly used in mental health research, reporting of equity-related demographics, which are relevant to representativeness, is inconsistent. Gender and age were universally reported, but items such as race/ethnicity, occupation, SES, place of residence, and religion were often missing. Evidence that DCTs enhance inclusion of underserved groups was limited. Barriers to equitable participation included digital access, literacy, and participant burden, while enablers included therapist/navigator support and simplified onboarding

This review has several strengths. Unlike many others, it incorporated PPI from the outset, shaping the research focus and data extraction [96]. To our knowledge, it is the first systematic review to apply PROGRESS-Plus to DCTs in mental health and to compare sociodemographic representation against national statistics. Previous reviews have focused on reporting quality [10,97], whereas our review examined representativeness as a key indicator of equity in DCTs. It is also uniquely specific to digital/remotely delivered mental healthcare, a global priority, and included international studies, strengthening generalisability. Our structured approach identified gaps in reporting of disadvantage-related demographics and highlighted at-risk groups for exclusion. Consistent with earlier findings, we show that despite claims that DCTs support inclusive participation, marginalised groups, particularly by ethnicity and SES, remain underreported, with religion, disability, and residence, are often overlooked [98–101]. While digital literacy and comfort with telemedicine are improving [102,103], remote/digital methods may not be sufficient to achieve inclusion in mental health research without consideration to specific methodological design. Nonetheless, the conclusions of this review should be interpreted alongside its limitations, such as that the exclusion of non-English studies and pre-2020 trials. Furthermore, inconsistent reporting of variables like socioeconomic status and social capital hindered synthesis, and dynamic demographics such as employment and education limit direct comparison with national statistics. Additionally, the comparison to national statistics was constrained by how variables were defined by the census data, we have outlined these in supplementary materials (S1 Data, S4 Table). Therefore, comparisons with national statistics should be interpreted as proxies rather than exact benchmarks.

**Fig 2:**
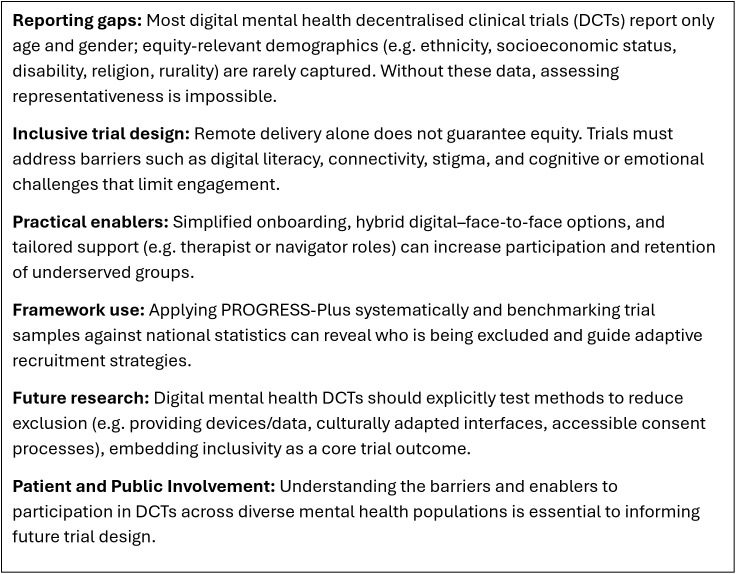
Implications for digital mental health research.

These findings have important implications for the design and implementation of digital mental health trials (Fig 2). For clinicians and service providers, the lack of consistent equity-relevant data reporting limits the ability to assess whether digital interventions are reaching and benefiting those most in need. Without robust demographic data, it is difficult to tailor interventions or address disparities in access and outcomes—particularly for groups historically underserved by mental health services [104,105]. For policymakers, the findings underscore the need for clearer guidance and accountability mechanisms to ensure equity is embedded in digital trial design and reporting. This includes mandating the collection and transparent reporting of PROGRESS-Plus variables, as recommended by the *PRISMA-Equity* extension and other equity-focused frameworks [31,33], to monitor progress towards inclusive research and to inform equitable policy decisions.

Methodologically, equity must be embedded from the outset. Researchers need support to design trials that promote inclusion across the entire lifecycle, not just at recruitment. While our review focused on baseline representation, we found limited reporting on attrition by sociodemographic group, highlighting the need to prioritise equitable participation as well as access. DCTs can expand access to innovations in mental healthcare—but only if equity is embedded in their design and infrastructure. Realising this potential demands collaboration across disciplines, including methodologists, clinicians, industry, and patient representatives. It also important to explore how different models of decentralisation (e.g., remote recruitment, virtual intervention delivery, and digital follow-up) affect participation, engagement, and outcomes across diverse groups. Inconsistent practices and limited reporting continue to obscure representativeness and limit generalisability.

Our findings suggest that digital access and literacy intersect closely with age, contributing to the underrepresentation of children, adolescents, and older adults across trials. Many DCTs required device ownership, stable internet access, or digital competence as eligibility criteria, and technical difficulties were frequently reported. These requirements may disproportionately exclude younger participants who depend on caregiver support, and older adults who often encounter greater challenges using digital platforms. Incorporating age-appropriate onboarding, simplified interfaces, and additional technical or navigational support may help reduce exclusion for these groups and promote more representative participation.

One practical step is to integrate PROGRESS-Plus variables into the study protocol from the outset, ensuring that place of residence, socioeconomic status, race/ethnicity, education, and social capital are captured systematically rather than selectively. Aligning ethnicity and SES indicators with national census categories or established deprivation indices would further improve comparability. In addition, DCTs would benefit from adopting clearer standards for collecting and reporting digital access and literacy, factors that are particularly relevant for decentralised designs but remain poorly documented. Simple, validated measures (e.g., device ownership, internet stability, digital self-efficacy scales) could be routinely included at baseline. Harmonised reporting of missing data and transparent documentation of how variables are operationalised (e.g., definition of SES categories) would also enhance interpretability.

## Conclusion

Although DCTs are often positioned as a way to democratise participation in mental health research, our findings indicate that equitable inclusion, as reflected by representativeness, remains underachieved. Socially disadvantaged groups continue to be inconsistently reported and persistently underrepresented. The absence of standardised, equity-focused data collection obscures who is included, who is excluded, and on what grounds. As digital mental health infrastructures expand, achieving equitable participation demands more than technological progress, it requires coordinated policy action, regulatory standards, and investment in inclusive research frameworks. Embedding equity as a core criterion in the design, funding, and evaluation of DCTs is essential to ensure that digital innovation advances, rather than reproduces, existing social and health inequalities.

## Supporting information

S1 PRISMA Equity Checklist. (DOCX)

S1 Data List of included papers. (DOCX)

S1 Table MEDLINE search strategy. (DOCX)

S2 Table Complete list of data extracted from included papers. (DOCX)

S3 Table Papers excluded at full-text review. (DOCX)

S4 Table Sociodemographic characteristics reported (4a) and key sources used (4b). S4a. Sociodemographic characteristics as reported by included studies. S4b Table. Sources used to determine sociodemographic characteristics of the country of study*.
(DOCX)

S5 Table Categorisations and data sources for socio-economic status (SES)/income. S5a Table. References used for calculating high, medium and low income earners by country. S5b Table. References used for determining studies included in the poverty comparisons.
(DOCX)
