# Supplementary material for: Who gets included? Equity in digital and decentralised mental health and neurodevelopmental trials: A systematic review
Source: PLOS Digit Health. 2026 Jun 8;5(6):e0001466. doi: 10.1371/journal.pdig.0001466 (PMC13245764; doi:10.1371/journal.pdig.0001466)
Supplement: S1 Data — (DOCX) [file pdig.0001466.s002.docx]

**S1 Data. List of included papers**

1. Andrews B, Klein B, Van Nguyen H, Corboy D, McLaren S, Watson S. Efficacy of a digital mental health biopsychosocial transdiagnostic intervention with or without therapist assistance for adults with anxiety and depression: adaptive randomized controlled trial. Journal of medical Internet research. 2023 Jun 12;25:e45135. ***[Ref 44]***
2. Backman A, Roll-Pettersson L, Mellblom A, Norman-Claesson E, Sundqvist E, Zander E, Vigerland S, Hirvikoski T. Internet-delivered psychoeducation (SCOPE) for transition-aged autistic youth: pragmatic randomized controlled trial. Journal of Medical Internet Research. 2024 Nov 28;26:e49305.3. ***[Ref 75]***
3. Bennett SD, Heyman I, Coughtrey AE, Varadkar S, Stephenson T, Shafran R. Telephone-guided self-help for mental health difficulties in neurological conditions: a randomised pilot trial. Archives of disease in childhood. 2021;106(9):862-7. ***[Ref 83]***
4. Bilan DS, Chicchi Giglioli IA, Cuesta P, Cañadas E, de Ramón I, Maestú F, et al. Decreased impulsiveness and MEG normalization after AI-digital therapy in ADHD children: a RCT. Npj Ment Health Res. 2025;4(1):1. ***[Ref 95]***
5. Carl JR, Miller CB, Henry AL, Davis ML, Stott R, Smits JAJ, et al. Efficacy of digital cognitive behavioral therapy for moderate-to-severe symptoms of generalized anxiety disorder: A randomized controlled trial. Depress Anxiety. 2020;37(12):1168-78. ***[Ref 40]***
6. Chan CS, Wong CYF, Yu BYM, Hui VKY, Ho FYY, Cuijpers P. Treating depression with a smartphone-delivered self-help cognitive behavioral therapy for insomnia: a parallel-group randomized controlled trial. Psychol Med. 2023;53(5):1799-813. ***[Ref 93]***
7. Chien C-W, Lin C-Y, Lai CYY, Graham F. Parent coaching to enhance community participation in young children with developmental disabilities: A pilot randomized controlled trial. Research in developmental disabilities. 2024;147(8709782, rid):104696. ***[Ref 94]***
8. Clark DM, Wild J, Warnock-Parkes E, Stott R, Grey N, Thew G, et al. More than doubling the clinical benefit of each hour of therapist time: a randomised controlled trial of internet cognitive therapy for social anxiety disorder. Psychological medicine. 2023;53(11):5022EP-32. ***[Ref 66]***
9. Creswell C, Taylor L, Giles S, Howitt S, Radley L, Whitaker E, et al. Digitally augmented, parent-led CBT versus treatment as usual for child anxiety problems in child mental health services in England and Northern Ireland: a pragmatic, non-inferiority, clinical effectiveness and cost-effectiveness randomised controlled trial. Lancet Psychiatry. 2024;11(3):193-209. ***[Ref 62]***
10. Döpfner M, Plück J, Rosenberger KD, Klemp MT, Mühlenmeister J, Wähnke L, et al. Efficacy of guided and unguided web-assisted self-help for parents of children with attention-deficit/hyperactivity disorder and oppositional defiant disorder: A three-arm randomized controlled trial. J Child Psychol Psychiatry. 2025. ***[Ref 91]***
11. Eto A, Endo A, Yoshida T, Seki Y, Taguchi K, Hongo M, et al. Videoconference-Based Cognitive Behavioral Therapy in Medication-Treated Adults with Attention-Deficit/Hyperactivity Disorder: A Randomized, Assessor-Blinded, Controlled Trial. Psychotherapy and psychosomatics. 2025(qha, 0024046):1-25. ***[Ref 77]***
12. Eylem O, van Straten A, de Wit L, Rathod S, Bhui K, Kerkhof AJFM. Reducing suicidal ideation among Turkish migrants in the Netherlands and in the UK: the feasibility of a randomised controlled trial of a guided online intervention. Pilot and Feasibility Studies. 2021;7(1):30. ***[Ref 41]***
13. Fatouros P, Tsirmpas C, Andrikopoulos D, Kaplow S, Kontoangelos K, Papageorgiou C. Randomized controlled study of a digital data driven intervention for depressive and generalized anxiety symptoms. NPJ Digit Med. 2025;8(1):113. ***[Ref 78]***
14. Felder JN, Epel ES, Neuhaus J, Krystal AD, Prather AA. Efficacy of Digital Cognitive Behavioral Therapy for the Treatment of Insomnia Symptoms Among Pregnant Women: A Randomized Clinical Trial. JAMA Psychiatry. 2020;77(5):484-92. ***[Ref 37]***
15. Felder JN, Epel ES, Neuhaus J, Krystal AD, Prather AA. Randomized controlled trial of digital cognitive behavior therapy for prenatal insomnia symptoms: effects on postpartum insomnia and mental health. Sleep. 2022;45(2). ***[Ref 38]***
16. Grenier-Martin J, Rivard M, Patel S, Lanovaz MJ, Lefebvre C. Randomized controlled trial on an online training to support caregivers of young children with intellectual and developmental disability managing problem behaviors at home. Journal of Child and Family Studies. 2022;31(12):3485-97. ***[Ref 69]***
17. Guzick AG, Schneider SC, Kook M, Greenberg R, Perozo-Garcia A, Lee MP, et al. Internet-based, parent-led cognitive behavioral therapy for autistic youth with anxiety-related disorders: A randomized trial comparing email vs. telehealth support. Behav Res Ther. 2024;183:104639. ***[Ref 52]***
18. Guzick AG, Schneider SC, Perozo Garcia AB, Kook M, Greenberg RL, Riddle D, et al. Development and pilot testing of internet-delivered, family-based cognitive behavioral therapy for anxiety and obsessive-compulsive disorders in autistic youth. Journal of Obsessive-Compulsive and Related Disorders. 2023;37. ***[Ref 60]***
19. Hall CL, Bergin ADG, Rennick-Egglestone S. Research Into Digital Health Intervention for Mental Health: 25-Year Retrospective on the Ethical and Legal Challenges. Journal of Medical Internet Research. 2024;26:e58939. ***[Ref 39]***
20. Hartley M, Dorstyn D, Due C. Challenges encountered with a mindfulness app: Lessons learnt from a pilot randomized trial involving caregivers and individuals with autism. Research in Autism Spectrum Disorders. 2022;96. ***[Ref 86]***
21. Haun JN, Venkatachalam HH, Fowler CA, Alman AC, Ballistrea LM, Schneider T, et al. Mobile and Web-Based Partnered Intervention to Improve Remote Access to Pain and Posttraumatic Stress Disorder Symptom Management: Recruitment and Attrition in a Randomized Controlled Trial. Journal of Medical Internet Research. 2023;25(1):e49678. ***[Ref 42]***
22. Heller HM, Hoogendoorn AW, Honig A, Broekman BFP, van Straten A. The Effectiveness of a Guided Internet-Based Tool for the Treatment of Depression and Anxiety in Pregnancy (MamaKits Online): Randomized Controlled Trial. Journal of medical Internet research. 2020;22(3):e15172. ***[Ref 49]***
23. He Y, Yang L, Zhu X, Wu B, Zhang S, Qian C, et al. Mental Health Chatbot for Young Adults With Depressive Symptoms During the COVID-19 Pandemic: Single-Blind, Three-Arm Randomized Controlled Trial. J Med Internet Res. 2022;24(11):e40719. ***[Ref 43]***
24. Hoffmann D, Rask CU, Hedman-Lagerlof E, Jensen JS, Frostholm L. Efficacy of internet-delivered acceptance and commitment therapy for severe health anxiety: results from a randomized, controlled trial. Psychological medicine. 2021;51(15):2685EP-95. ***[Ref 72]***
25. Hollis C, Hall CL, Jones R, Marston L, Novere ML, Hunter R, et al. Therapist-supported online remote behavioural intervention for tics in children and adolescents in England (ORBIT): a multicentre, parallel group, single-blind, randomised controlled trial. The lancet Psychiatry. 2021;8(10):871-82. ***[Ref 63]***
26. Huberty J, Puzia ME, Green J, Vlisides-Henry RD, Larkey L, Irwin MR, et al. A mindfulness meditation mobile app improves depression and anxiety in adults with sleep disturbance: Analysis from a randomized controlled trial. Gen Hosp Psychiatry. 2021;73:30-7. ***[Ref 53]***
27. Jamali AR, Zarei MA, Sanjari MA, AkbarFahimi M, Saneii SH. Randomized controlled trial of occupation performance coaching for families of children with autism spectrum disorder by means of telerehabilitation. The British Journal of Occupational Therapy. 2022;85(5):308-15. ***[Ref 80]***
28. Jent JF, Rothenberg WA, Weinstein A, Stokes J, Barnett M, Srivatsa N, et al. Comparing Traditional and Ebook-Augmented Parent-Child Interaction Therapy (PCIT): A Randomized Control Trial of Pocket PCIT. Behavior therapy. 2021;52(6):1311-24. ***[Ref 51]***
29. Kalmbach DA, Cheng P, O'Brien LM, Swanson LM, Sangha R, Sen S, et al. A randomized controlled trial of digital cognitive behavioral therapy for insomnia in pregnant women. Sleep Med. 2020;72:82-92. ***[Ref 54]***
30. Kandola A, Edwards K, Muller MA, Dührkoop B, Hein B, Straatman J, et al. Digitally managing depression: A fully remote randomised attention-placebo controlled trial. Digit Health. 2024;10:20552076241260409. ***[Ref 84]***
31. Kenworthy L, Childress D, Armour AC, Verbalis A, Zhang A, Troxel M, et al. Leveraging technology to make parent training more accessible: Randomized trial of in-person versus online executive function training for parents of autistic children. Autism : the international journal of research and practice. 2023;27(3):616-28. ***[Ref 61]***
32. Kwon SY, Seo G, Jang M, Shin H, Choi W, Lim YB, et al. The Effect of Mobile Neurofeedback Training in Children with Attention Deficit Hyperactivity Disorder: A Randomized Controlled Trial. Clin Psychopharmacol Neurosci. 2024;22(1):67-78. ***[Ref 79]***
33. Lewis S, Rinehart N, Mantilla A, Alvares G, Hiscock H, Marks D, et al. A pilot randomised controlled trial of a telehealth-delivered brief 'Sleeping Sound Autism' intervention for autistic children. Sleep medicine. 2024;124(100898759):162-73. ***[Ref 48]***
34. Lindgren S, Wacker D, Schieltz K, Suess A, Pelzel K, Kopelman T, et al. A Randomized Controlled Trial of Functional Communication Training via Telehealth for Young Children with Autism Spectrum Disorder. J Autism Dev Disord. 2020;50(12):4449-62. ***[Ref 81]***
35. Lippke S, Gao L, Keller FM, Becker P, Dahmen A. Adherence with online therapy vs face-to-face therapy and with online therapy vs care as usual: Secondary analysis of two randomized controlled trials. Journal of Medical Internet Research. 2021;23(11):e31274. ***[Ref 70]***
36. Malarkey ME, Fu AJ, Mannan N, Shaw OM, Haight TJ, Cota MR, et al. Internet-Guided Cognitive Behavioral Therapy for Insomnia Among Patients With Traumatic Brain Injury: A Randomized Clinical Trial. JAMA Network Open. 2024;7(7):e2420090. ***[Ref 55]***
37. March S, Spence SH, Myers L, Ford M, Smith G, Donovan CL. Integrating Videoconferencing Therapist Guidance Into Stepped Care Internet-Delivered Cognitive Behavioral Therapy for Child and Adolescent Anxiety: Noninferiority Randomized Controlled Trial. JMIR Ment Health. 2025;12:e57405. ***[Ref 45]***
38. March S, Spence SH, Myers L, Ford M, Smith G, Donovan CL. Stepped-care versus therapist-guided, internet-based cognitive behaviour therapy for childhood and adolescent anxiety: A non-inferiority trial. Internet Interv. 2023;34:100675. ***[Ref 46]***
39. McCloud T, Jones R, Lewis G, Bell V, Tsakanikos E. Effectiveness of a Mobile App Intervention for Anxiety and Depression Symptoms in University Students: Randomized Controlled Trial. JMIR Mhealth Uhealth. 2020;8(7):e15418. ***[Ref 85]***
40. McLellan LF, Woon S, Hudson JL, Lyneham HJ, Karin E, Rapee RM. Treating child anxiety using family-based internet delivered cognitive behavior therapy with brief therapist guidance: A randomized controlled trial. J Anxiety Disord. 2024;101:102802. ***[Ref 47]***
41. Mechler J, Lindqvist K, Carlbring P, Topooco N, Falkenström F, Lilliengren P, et al. Therapist-guided internet-based psychodynamic therapy versus cognitive behavioural therapy for adolescent depression in Sweden: a randomised, clinical, non-inferiority trial. Lancet Digit Health. 2022;4(8):e594-e603. ***[Ref 88]***
42. Moshe I, Terhorst Y, Paganini S, Schlicker S, Pulkki-Raback L, Baumeister H, et al. Predictors of Dropout in a Digital Intervention for the Prevention and Treatment of Depression in Patients with Chronic Back Pain: Secondary Analysis of Two Randomized Controlled Trials. Journal of Medical Internet Research. 2022;24(8):e38261. ***[Ref 92]***
43. Murray G, Thomas N, Michalak EE, Jones SH, Lapsley S, Bowe SJ, et al. Mindfulness-Based Online Intervention to Improve Quality of Life in Late-Stage Bipolar Disorder: A Randomized Clinical Trial. Journal of Consulting and Clinical Psychology. 2021;89(10):830EP-44. ***[Ref 87]***
44. Nardi W, Roy A, Dunsiger S, Brewer J. Analyzing the Impact of Mobile App Engagement on Mental Health Outcomes: Secondary Analysis of the Unwinding Anxiety Program. Journal of Medical Internet Research. 2022;24(8):e33696. ***[Ref 73]***
45. Nissling L, Weineland S, Vernmark K, Radvogin E, Engström AK, Schmidt S, et al. Effectiveness of and processes related to internet-delivered acceptance and commitment therapy for adolescents with anxiety disorders: a randomized controlled trial. Res Psychother. 2023;26(2). ***[Ref 76]***
46. Nordh M, Wahlund T, Jolstedt M, Sahlin H, Bjureberg J, Ahlen J, et al. Therapist-Guided Internet-Delivered Cognitive Behavioral Therapy vs Internet-Delivered Supportive Therapy for Children and Adolescents With Social Anxiety Disorder: A Randomized Clinical Trial. JAMA Psychiatry. 2021;78(7):705-13. ***[Ref 74]***
47. Ong LE, Speicher S, Villasenor D, Kim J, Jacobs A, Macia KS, et al. Brief Peer-Supported Web-Based Skills Training in Affective and Interpersonal Regulation (BPS webSTAIR) for Trauma-Exposed Veterans in the Community: Randomized Controlled Trial. Journal of Medical Internet Research. 2024;26:e52130. ***[Ref 56]***
48. Piscitello J, Leon D, Ramos MC, Robertson EL, Altszuler AR, Pelham WE. A randomized controlled trial of a virtually delivered group parent training for parents of children with ADHD during an intensive summer treatment program. Evidence-Based Practice in Child and Adolescent Mental Health. 2025 Apr 3;10(2):450-64. ***[Ref 57]***
49. Possemato K, Wu J, Greene C, MacQueen R, Blonigen D, Wade M, et al. Web-Based Problem-solving Training With and Without Peer Support in Veterans With Unmet Mental Health Needs: Pilot Study of Feasibility, User Acceptability, and Participant Engagement. Journal of Medical Internet Research. 2022;24(1):e29559. ***[Ref 58]***
50. Richards D, Enrique A, Eilert N, Franklin M, Palacios J, Duffy D, et al. A pragmatic randomized waitlist-controlled effectiveness and cost-effectiveness trial of digital interventions for depression and anxiety. NPJ Digit Med. 2020;3:85. ***[Ref 64]***
51. Sabri B, Perrin N, Hagos M. The Being Safe, Health and Positively Empowered Pilot Randomized Controlled Trial: A Digital Multicomponent Intervention for Immigrant Women With Cumulative Exposures to Violence. Cultural Diversity and Ethnic Minority Psychology. 2025;31(2):271EP-84. ***[Ref 82]***
52. Sayal K, Wyatt L, Partlett C, Ewart C, Bhardwaj A, Dubicka B, et al. The clinical and cost effectiveness of a STAndardised DIagnostic Assessment for children and adolescents with emotional difficulties: the STADIA multi-centre randomised controlled trial. J Child Psychol Psychiatry. 2025;66(6):805-20. ***[Ref 65]***
53. Segal ZV, Dimidjian S, Beck A, Boggs JM, Vanderkruik R, Metcalf CA, et al. Outcomes of Online Mindfulness-Based Cognitive Therapy for Patients With Residual Depressive Symptoms: A Randomized Clinical Trial. JAMA Psychiatry. 2020;77(6):563-73. ***[Ref 59]***
54. Seo JM, Kim SJ, Na H, Kim JH, Lee H. Effectiveness of a Mobile Application for Postpartum Depression Self-Management: Evidence from a Randomised Controlled Trial in South Korea. Healthcare (Basel). 2022;10(11). ***[Ref 71]***
55. Sun Y, Li Y, Wang J, Chen Q, Bazzano AN, Cao F. Effectiveness of smartphone-based mindfulness training on maternal perinatal depression: Randomized controlled trial. Journal of Medical Internet Research. 2021;23(1):e23410. ***[Ref 67]***
56. Tan S, Ismail MAB, Daud TIM, Hod R, Ahmad N. A randomized controlled trial on the effect of smartphone-based mental health application among outpatients with depressive and anxiety symptoms: A pilot study in Malaysia. Indian Journal of Psychiatry. 2023;65(9):934EP-40. ***[Ref 50]***
57. Tan Y, Lyu R, Lu S. Reducing parenting stress in Chinese parents of children with learning disabilities with a mindful parenting program: A randomized controlled trial. Research in developmental disabilities. 2024;151(8709782, rid):104794. ***[Ref 89]***
58. Wong VW, Ho FY, Shi NK, Tong JT, Chung KF, Yeung WF, et al. Smartphone-delivered multicomponent lifestyle medicine intervention for depressive symptoms: A randomized controlled trial. J Consult Clin Psychol. 2021;89(12):970-84. ***[Ref 68]***
59. Wu Y, Li X, Zhou Y, Gao R, Wang K, Ye H, et al. Efficacy and Cost-Effectiveness Analysis of Internet-Based Cognitive Behavioral Therapy for Obsessive-Compulsive Disorder: Randomized Controlled Trial. Journal of Medical Internet Research. 2023;25:e41283. ***[Ref 90]***
