# Supplementary material for: Who gets included? Equity in digital and decentralised mental health and neurodevelopmental trials: A systematic review
Source: PLOS Digit Health. 2026 Jun 8;5(6):e0001466. doi: 10.1371/journal.pdig.0001466 (PMC13245764; doi:10.1371/journal.pdig.0001466)
Supplement: S1 Table — (DOCX) [file pdig.0001466.s003.docx]

**S1 Table. MEDLINE search strategy**

| 1. | exp Telemedicine/ |
| --- | --- |
| 2. | ((decentrali#ed or remote* or virtual* or online or digital* or hybrid or "internet-based" or "technology-enabled" or "app-based") AND (trial* or intervention* or therap* or care or monitor* or assess* or diagnos*)).mp. |
| 3. | (telemed* or tele-med* or telehealth or tele-health or teletherap* or tele-therap* or ehealth or e-health or mhealth or m-health or "mobile health" or "digital health").mp. |
| 4. | (Randomised controlled trial or random* or controlled clinical trial or trial or RCT).mp. |
| 5. | 1 OR 2 OR 3 OR 4 |
| 6. | Mental Health/ |
| 7. | exp Mental Disorders/ |
| 8. | ((mental* or psychiatric* or psychological* or psychotic*) AND (health* or ill* or unwell or disorder*)).mp. |
| 9. | (depress* or anxi* or "bipolar disorder*" or schizophreni* or "post-traumatic stress" or PTSD or "eating disorder*" or "obsessive-compulsive disorder*" or OCD or "mood disorder*" or "behavio?ral health").mp. |
| 10. | 6 OR 7 OR 8 OR 9 |
| 11. | Exp Neurodevelopmental Disorders/ |
| 12. | ("neurodevelopmental disorders" or "intellectual disability" or "autism" or "attention deficit disorder" or "disruptive behavio*" or hyperactivity).mp. |
| 13. | 11 OR 12 |
| 14. | exp Health Services Accessibility/ |
| 15. | exp Minority Groups/ |
| 16. | exp Ethnic Groups/ |
| 17. | exp Disabled Persons/ |
| 18. | (underrepresent* or "under represent*" or underserved or "under served" or unrepresent* or "not represent*" or "low* representation" or "lack of representation" or exclusion or excluded).mp. |
| 19. | ((ethnic or racial or race* or indigenous or relig* or marginali#ed or demographic or vulnerable) adj3 (group* or minorit* or population* or communit*)).mp. |
| 20. | (inclusi* or divers* or "health equity" or "access to care" or unequal or inequit* or inequalit*).mp. |
| 21. | ("person* of colo?r" or "people of colo?r" or "low* income*" or poor* or poverty or unemploy* or "socio-economic status" or "socio-economic* disadvantage*" or disab* or "limited mobility" or "restricted mobility" or "physical* impair*" or "education* level*" or "education* disadvantage*" or "digital literac*" or "digital divide" or "remote area*" or rural or pregnan*).mp. |
| 22. | 14 OR 15 OR 16 OR 17 OR 18 OR 19 OR 20 OR 21 |
| 23. | (barrier* or facilitat* or recruit* or retain* or retention or dropout* or "drop* out" or engag* or disengag* or inhibit* or difficult* or constrain* or limit* or challeng* or obstacle* or problem* or interfer* or obstruct* or restrain* or restrict* or disincentiv* or "low* participation" or "low* engagement" or discourag*).mp. |
| 24. | 5 AND 10 AND 13 AND 22 AND 23 |
| 25. | Limit 24 to yr=”2020-Current” |
