# Supplementary material for: Who gets included? Equity in digital and decentralised mental health and neurodevelopmental trials: A systematic review
Source: PLOS Digit Health. 2026 Jun 8;5(6):e0001466. doi: 10.1371/journal.pdig.0001466 (PMC13245764; doi:10.1371/journal.pdig.0001466)
Supplement: S2 Table — (DOCX) [file pdig.0001466.s004.docx]

**S2 Table. Complete list of data extracted from included papers**

| 1 | Author/Year |
| --- | --- |
| 2 | Country |
| 3 | Study design |
| 4 | Sample size (baseline; as reported in demographics table) |
| 5 | Mental health/neurodevelopmental condition |
| 6 | Full of hybrid remote |
| 7 | Remote recruitment strategies used (Y/N/not clear) |
| 8 | Screening and/or eligibility remote (Y/N/not clear) |
| 9 | Assessments/follow-up remote (Y/N/not clear) |
| 10 | Type of intervention (virtual reality, digital, web-based, wearable device, telehealth) |
| 11 | Intervention setting (e.g., home, community) |
| 12 | Issues related to inclusivity if mentioned |
| 13 | Comparison of the study population to other populations in terms of representativeness (e.g., general population, demographics within a specific condition) |
| 14 | Barriers to participant/engagement |
| 15 | Facilitators to participant/engagement |
| 16 | Drop out by demographics |
| 17 | Digital literacy or access reported (Y/N) |
| 18 | If digital literacy or access reported, what information? |
| 19 | Equity reporting (PROGRESS-PLUS): Place (e.g., rural, urban, LMIC, HMIC) |
| 20 | Equity reporting (PROGRESS-PLUS): Race/ethnicity (e.g., Asian, Black, Multiple, White, other) |
| 21 | Equity reporting (PROGRESS-PLUS): Occupational level (e.g., employed, not employed) |
| 22 | Equity reporting (PROGRESS-PLUS): Gender and sex (e.g., male, female, non-binary, trans, other) |
| 23 | Equity reporting (PROGRESS-PLUS): Religion (e.g., Christian, Catholic, Buddhist, Hindu, Jewish, Muslim, Sikh, other) |
| 24 | Equity reporting (PROGRESS-PLUS): Education (e.g., early, primary, secondary, further, higher) |
| 25 | Equity reporting (PROGRESS-PLUS): Socioeconomic status (e.g., income) |
| 26 | Equity reporting (PROGRESS-PLUS): Social capital (e.g., relationships/network) |
| 27 | Equity reporting (PROGRESS-PLUS): Age |
