# Supplementary material for: Who gets included? Equity in digital and decentralised mental health and neurodevelopmental trials: A systematic review
Source: PLOS Digit Health. 2026 Jun 8;5(6):e0001466. doi: 10.1371/journal.pdig.0001466 (PMC13245764; doi:10.1371/journal.pdig.0001466)
Supplement: S3 Table — (DOCX) [file pdig.0001466.s005.docx]

**S3 Table. Papers excluded at full-text review.**

| **Title** | **Authors** | **Year** | **Journal** | **Exclusion reason** |
| --- | --- | --- | --- | --- |
| Internet-delivered Parent-Child Interaction Therapy and Sleep Quality in Children With Developmental Delay: Examining the Mediating Role of Bedtime Resistance Behaviors. | Warner, Morganne; Gillenson, Caroline J; Parent, Justin; Comer, Jonathan S; Bagner, Daniel M | 2025 | Journal of developmental and behavioral pediatrics | No other elements of trial design apart from intervention are online |
| Enhancing task performance in adults with intellectual disability through modified goal management training and assistive technology with errorless learning: A randomized controlled trial. | Ertas-Spantgar, Funda; Hildebrandt, Helmut; Gabel, Alexander; Schiering, Ina; Muller, Sandra Verena | 2025 | Neuropsychological rehabilitation | No confirmed mental health or neurodevelopmental diagnosis |
| An online, father-inclusive parenting intervention for reducing child conduct problems: a randomised controlled trial of family man. | Tully, Lucy A; Turnell, Adrienne I; Morgan, Bronte G; Hawes, David J; Anderson, Jenny; Kean, Anna; Dadds, Mark R | 2025 | Journal of child psychology and psychiatry, and allied disciplines | No confirmed mental health or neurodevelopmental diagnosis |
| Clinical effectiveness of an online psychoeducational and psychotherapeutic programme for caregivers of children newly diagnosed as autistic: a parallel, assessor-masked, randomised controlled trial in the UK (REACH-ASD). | Leadbitter, Kathy; Langhorne, Sophie; Smallman, Richard; Chu, Petrina; Ellis, Ceri; Harrison, Louisa; Hutton, Tessa; Butter, Charlotte; Goldie, Caitlin; James, Kirsty; Hackett, Latha; Dunkerley, Alison; Bee, Penny; Shields, Gemma; Davies, Linda; Emsley, Richard; Green, Jonathan | 2025 | The lancet psychiatry | No confirmed mental health or neurodevelopmental diagnosis |
| Efficacy of internet-based cognitive behavioral therapy for medicated adults with attention-deficit/hyperactivity disorder (ADHD): A randomized controlled trial. | Zhang, Shi-Yu; Pan, Mei-Rong; Zhang, Li-Qian; Li, Hai-Mei; Zhao, Meng-Jie; Dong, Min; Si, Fei-Fei; Liu, Lu; Wang, Yu-Feng; Qian, Qiu-Jin | 2025 | Psychiatry research | No other elements of trial design apart from intervention are online |
| Supporting young siblings of children with intellectual disabilities and/or visual impairments with the serious game 'Broodles': A mixed methods randomized controlled trial. | Veerman, Linda K M; Willemen, Agnes M; Derks, Suzanne D M; Brouwer-van Dijken, Anjet A J; Sterkenburg, Paula S | 2025 | Research in developmental disabilities | No confirmed mental health or neurodevelopmental diagnosis |
| The Effect of a Video-Based Game Exercise Program on Motor Skills, Proprioception, and Cognitive Functions in Individuals With Intellectual Disabilities. | Diril, Elif; Menek, Burak; Emir, Ahmet; Tarakci, Devrim; Tarakci, Ela | 2025 | Occupational therapy international | No other elements of trial design apart from intervention are online; |
| Automated Detection of Neurodevelopmental Disorders Using Face-to-Face Mobile Technology Among Typically Developing Greek Children: Randomized Controlled Trial. | Toki, Eugenia I; Zakopoulou, Victoria; Tatsis, Giorgos; Pange, Jenny | 2024 | JMIR formative research | No confirmed mental health or neurodevelopmental diagnosis |
| Process evaluation of a parenting intervention for pre-schoolers with intellectual disabilities who display behaviours that challenge in the UK. | Ondruskova, Tamara; Oulton, Kate; Royston, Royston; Hassiotis, Angela | 2024 | Journal of applied research in intellectual disabilities: JARID | Non-digital intervention; |
| Family engagement in a behavioral parenting intervention: A randomized comparison of telehealth versus office-based treatment formats. | Sanchez, Amanda L; Javadi, Natalie; Comer, Jonathan S | 2024 | Journal of consulting and clinical psychology | Not emotional/behavioural related outcomes; |
| Technology-Enhanced BPT for Early-Onset Behavior Disorders: Improved Outcomes for Children With Co-Occurring Internalizing Symptoms. | Parent, Justin; Highlander, April; Loiselle, Raelyn; Yang, Yexinyu; McKee, Laura G; Forehand, Rex; Jones, Deborah J | 2024 | Journal of clinical child and adolescent psychology : the official journal for the Society of Clinical Child and Adolescent Psychology, American Psychological Association, Division 53 | Before 2020; |
| Digital adaptation of the Standing up for Myself intervention in young people and adults with intellectual disabilities: the STORM feasibility study. | Scior, Katrina; Richardson, Lisa; Randell, Elizabeth; Osborne, Michaela; Bird, Harriet; Ali, Afia; Bonin, Eva-Maria; Brown, Adrian; Brown, Celia; Burke, Christine-Koulla; Bush, Lisa; Crabtree, Jason; Davies, Karuna; Davies, Paul; Gillespie, David; Jahoda, Andrew; Johnson, Sean; Hastings, Richard; Kerr, Laura; McNamara, Rachel; Menzies, Jane; Roche, Harry; Wright, Melissa; Zhang, Kyann | 2024 | Public health research | Not RCT (or based on trial); |
| Effectiveness of the mentalisation-based serious game 'You & I' for adults with mild to borderline intellectual disabilities: A randomised controlled trial. | Derks, Suzanne D M; Willemen, Agnes M; Wouda, Mirjam; Sterkenburg, Paula S | 2024 | Journal of applied research in intellectual disabilities | No confirmed mental health or neurodevelopmental diagnosis |
| Implementing school-based cognitive behavior therapy for anxiety in students with autism or suspected autism via a train-the-trainer approach: Results from a clustered randomized trial. | Reaven, Judy; Pickard, Katherine; Meyer, Allison T; Hayutin, Lisa; Middleton, Caitlin; Reyes, Nuri M; Tanda, Tanea; Stahmer, Aubyn; Blakeley-Smith, Audrey; Boles, Richard E | 2024 | Autism: the international journal of research and practice | No confirmed mental health or neurodevelopmental diagnosis; |
| Moderators of the Effects of a Digital Parenting Intervention on Child Conduct and Emotional Problems Implemented During the COVID-19 Pandemic: Results From a Secondary Analysis of Data From the Supporting Parents and Kids Through Lockdown Experiences (SP | Pokorna, Nikola; Palmer, Melanie; Pearson, Oliver; Beckley-Hoelscher, Nicholas; Shearer, James; Kostyrka-Allchorne, Katarzyna; Robertson, Olly; Koch, Marta; Slovak, Petr; Day, Crispin; Byford, Sarah; Waite, Polly; Creswell, Cathy; Sonuga-Barke, Edmund J S; Goldsmith, Kimberley | 2024 | JMIR pediatrics and parenting | No confirmed mental health or neurodevelopmental diagnosis; |
| Putting the usability of wearable technology in forensic psychiatry to the test: a randomized crossover trial. | de Looff, Peter C; Noordzij, Matthijs L; Nijman, Henk L I; Goedhard, Laurette; Bogaerts, Stefan; Didden, Robert | 2024 | Frontiers in psychiatry | Not emotional/behavioural related outcomes; |
| Randomised controlled feasibility study protocol of the Carers-ID online intervention to support the mental health of family carers of people with intellectual disabilities. | Linden, Mark A; Leonard, Rachel; Forbes, Trisha; Brown, Michael; Marsh, Lynne; Todd, Stuart; Hughes, Nathan; Truesdale, Maria | 2024 | Pilot and feasibility studies | protocol; |
| The Effects of Young Children's Callous-Unemotional Traits on Behaviorally Observed Outcomes in Standard and Technology-Enhanced Behavioral Parent Training. | Parent, Justin; Jones, Deborah J; DiMarzio, Karissa; Yang, Yexinyu; Wright, Kat L; Sullivan, Alexandra D W; Forehand, Rex | 2023 | Research on child and adolescent psychopathology | Before 2020; |
| Social COmmunication Program supported by E-health (SCOPE) for infants and toddlers at elevated likelihood of autism spectrum disorder: study design of a cluster randomized controlled trial. | Snijder, Michelle I J; Dietz, Claudine; van Andel, Mieke; Ruiter, Emilie L M; Buitelaar, Jan K; Oosterling, Iris J | 2022 | BMC psychiatry | protocol; |
| A modified video-feedback intervention for carers of foster children aged 6 years and under with reactive attachment disorder: a feasibility study and pilot RCT. | Oliveira, Paula; Stevens, Eloise; Barge, Lydia; Comyn, Julie; Langley, Kirsty; Ramchandani, Paul; Wright, Barry; Woolgar, Matt; Kennedy, Eilis; Byford, Sarah; Shearer, James; Scott, Stephen; Barlow, Jane; Glaser, Danya; Senior, Rob; Fonagy, Peter; Fearon, Pasco | 2022 | Health technology assessment (Winchester, England) | No other elements of trial design apart from intervention are online; |
| Where Exactly Is the Therapist in Virtual Reality and Game-Based Rehabilitation Applications? A Randomized Controlled Trial in Children with Specific Learning Disability. | Kose, Barkin; Temizkan, Ege; Aran, Orkun Tahir; Galipoglu, Hasan; Torpil, Berkan; Pekcetin, Serkan; Karabulut, Erdem; Sahin, Sedef | 2022 | Games for health journal | Not emotional/behavioural related outcomes; |
| Rigorous Qualitative Research Involving Data Collected Remotely From People With Communication Disorders: Experience From a Telerehabilitation Trial. | Anglade, Carole; Tousignant, Michel; Gaboury, Isabelle | 2022 | Neurorehabilitation and neural repair | Not RCT (or based on trial) |
| A Distance-Delivered Social Skills Program for Young Adults with Williams Syndrome: Evaluating Feasibility and Preliminary Efficacy. | Fisher, Marisa H; Kammes, Rebecca R; Black, Rhonda S; Houck, Kristin; Cwiakala, Katie | 2022 | Journal of autism and developmental disorders | No confirmed mental health or neurodevelopmental diagnosis; |
| Efficacy and safety of balovaptan for socialisation and communication difficulties in autistic adults in North America and Europe: a phase 3, randomised, placebo-controlled trial. | Jacob, Suma; Veenstra-VanderWeele, Jeremy; Murphy, Declan; McCracken, James; Smith, Janice; Sanders, Kevin; Meyenberg, Christoph; Wiese, Thomas; Deol-Bhullar, Gurpreet; Wandel, Christoph; Ashford, Elizabeth; Anagnostou, Evdokia | 2022 | The lancet psychiatry | Non-digital intervention; |
| A randomized controlled trial of technology-enhanced behavioral parent training: sustained parent skill use and child outcomes at follow-up. | Parent, Justin; Anton, Margaret T; Loiselle, Raelyn; Highlander, April; Breslend, Nicole; Forehand, Rex; Hare, Megan; Youngstrom, Jennifer K; Jones, Deborah J | 2022 | Journal of child psychology and psychiatry, and allied disciplines | No confirmed mental health or neurodevelopmental diagnosis; |
| Effectiveness of the online mindset intervention 'The Growth Factory' for adolescents with intellectual disabilities. | Verberg, Fenneke; Helmond, Petra; Otten, Roy; Overbeek, Geertjan | 2022 | Journal of applied research in intellectual disabilities | No other elements of trial design apart from intervention are online; |
| A manualised weight management programme for adults with mild-moderate intellectual disabilities affected by excess weight: A randomised controlled feasibility trial (Shape Up-LD). | Lally, Phillippa; Beeken, Rebecca J; Wilson, Rose; Omar, Rumana; Hunter, Rachael; Fovargue, Sally; Anderson, Diana; King, Michael; Hassiotis, Angela; Croker, Helen | 2022 | Journal of applied research in intellectual disabilities | Non-digital intervention; |
| Stress in teachers of children with neuro-developmental disorders: Effect of blended rational emotive behavioral therapy. | Obiweluozo, Patience E; Dike, Ibiwari C; Ogba, Francisca N; Elom, Chinyere O; Orabueze, Florence O; Okoye-Ugwu, Stella; Ani, Casmir Kc; Onu, Augustine O; Ukaogo, Victor; Obayi, Loveline N; Abonyi, Sunday E; Onu, Janefrancis; Omenma, Zippora O; Okoro, Ifeanyichukwu D; Eze, Angela; Igu, Ntasiobi Cn; Onuigbo, Liziana N; Umeano, Elsie C; Onyishi, Charity N | 2021 | Science progress | No confirmed mental health or neurodevelopmental diagnosis; |
| A Brief Home-Based Parenting Intervention to Reduce Behavior Problems in Young Children: A Pragmatic Randomized Clinical Trial. | O'Farrelly, Christine; Watt, Hilary; Babalis, Daphne; Bakermans-Kranenburg, Marian J; Barker, Beth; Byford, Sarah; Ganguli, Poushali; Grimas, Ellen; Iles, Jane; Mattock, Holly; McGinley, Julia; Phillips, Charlotte; Ryan, Rachael; Scott, Stephen; Smith, Jessica; Stein, Alan; Stevens, Eloise; van IJzendoorn, Marinus H; Warwick, Jane; Ramchandani, Paul G | 2021 | JAMA pediatrics | No other elements of trial design apart from intervention are online; |
| Virtual interview training for autistic transition age youth: A randomized controlled feasibility and effectiveness trial. | Smith, Matthew J; Sherwood, Kari; Ross, Brittany; Smith, Justin D; DaWalt, Leann; Bishop, Lauren; Humm, Laura; Elkins, Jeff; Steacy, Chris | 2021 | Autism: the international journal of research and practice | Not emotional/behavioural related outcomes; |
| Challenges to conducting randomised controlled trials with adults with intellectual disabilities: Experiences of international experts. | Mulhall, Peter; Taggart, Laurence; McAloon, Toni; Coates, Vivien | 2021 | Journal of applied research in intellectual disabilities | Non-digital intervention; |
| An eHealth insomnia intervention for children with neurodevelopmental disorders: Results of a usability study. | Tan-MacNeill, Kim M; Smith, Isabel M; Weiss, Shelly K; Johnson, Shannon A; Chorney, Jill; Constantin, Evelyn; Shea, Sarah; Hanlon-Dearman, Ana; Brown, Cary A; Godbout, Roger; Ipsiroglu, Osman; Reid, Graham J; Corkum, Penny V | 2020 | Research in developmental disabilities | Not RCT (or based on trial); |
| Motor learning and transfer between real and virtual environments in young people with autism spectrum disorder: A prospective randomized cross over controlled trial. | de Moraes, Ibis Ariana Pena; Monteiro, Carlos Bandeira de Mello; Silva, Talita Dias da; Massetti, Thais; Crocetta, Tania Brusque; de Menezes, Lilian Del Ciello; Andrade, Gilda Pena de Rezende; Re, Alessandro Hervaldo Nicolai; Dawes, Helen; Coe, Shelly; Magalhaes, Fernando Henrique | 2020 | Autism research: official journal of the International Society for Autism Research | Not emotional/behavioural related outcomes; |
| Multisystemic therapy versus management as usual in the treatment of adolescent antisocial behaviour (START): 5-year follow-up of a pragmatic, randomised, controlled, superiority trial. | Fonagy, Peter; Butler, Stephen; Cottrell, David; Scott, Stephen; Pilling, Stephen; Eisler, Ivan; Fuggle, Peter; Kraam, Abdullah; Byford, Sarah; Wason, James; Smith, Jonathan A; Anokhina, Alisa; Ellison, Rachel; Simes, Elizabeth; Ganguli, Poushali; Allison, Elizabeth; Goodyer, Ian M | 2020 | The lancet psychiatry | No other elements of trial design apart from intervention are online; |
| Can an online exercise prescription tool improve adherence to home exercise programmes in children with cerebral palsy and other neurodevelopmental disabilities? A randomised controlled trial. | Johnson, Rowan W; Williams, Sian A; Gucciardi, Daniel F; Bear, Natasha; Gibson, Noula | 2020 | BMJ open | Not emotional/behavioural related outcomes; |
| Self-management training vs. neurofeedback interventions for attention deficit hyperactivity disorder: Results of a randomized controlled treatment study. | Korfmacher, Ann-Kathrin; Hirsch, Oliver; Chavanon, Mira-Lynn; Albrecht, Bjorn; Christiansen, Hanna | 2022 | Frontiers in Psychiatry | Non-digital intervention; |
| Cognitive Functional Remote Group intervention for adults with attention deficit hyperactivity disorder: A feasibility study. | Galili-Simhon, Shoval; Maeir, Adina | 2023 | The British Journal of Occupational Therapy | Not RCT (or based on trial); |
| Comparing telehealth to in-person early intervention: A quasi-experimental non-inferiority trial and cost-effectiveness analysis. | Sone, Bailey J; | 2024 | Dissertation Abstracts International Section A: Humanities and Social Sciences | Dissertation; |
| Pilot trial of a peer-to-peer psychoeducational intervention for parents of Black children awaiting a developmental evaluation. | Dababnah, Sarah; Reyes, Charina; Kim, Irang; Badawi, Deborah G; Chung, Yoonzie; Shaia, Wendy E | 2023 | Journal of Developmental and Behavioral Pediatrics | No confirmed mental health or neurodevelopmental diagnosis; |
| Efficacy and implementation of stress-reduction interventions for underserved families of autistic preschoolers across in-person and virtual modalities. | Fenning, Rachel M; Neece, Cameron L; Sanner, Catherine M; Morrell, Holly E. R; | 2024 | Mindfulness | No confirmed mental health or neurodevelopmental diagnosis; |
| Evaluation of the effects of the Online and Applied System for Intervention Skills (OASIS) with parents of children with ASD using a randomized waitlist control trial. | Heitzman-Powell, Linda; Buzhardt, Jay; Zhang, E; Barr, Jessica | 2023 | Behavior Analysis: Research and Practice | Non-digital intervention; |
| "Therapist-supported online remote behavioural intervention for tics in children and adolescents in England (ORBIT): A multicentre, parallel group, singleblind, randomised controlled trial": Correction. | Hollis, Chris; Hall, Charlotte L; Jones, Rebecca; Marston, Louise; Novere, Marie Le; Hunter, Rachael; Brown, Beverley J; Sanderson, Charlotte; Andren, Per; Bennett, Sophie D; Chamberlain, Liam R; Davies, E. Bethan; Evans, Amber; Kouzoupi, Natalia; McKenzie, Caitlin; Heyman, Isobel; Khan, Kareem; Kilgariff, Joseph; Glazebrook, Cristine; Mataix-Cols, David; Murphy, Tara; Serlachius, Eva; Murray, Elizabeth | 2022 | The Lancet Psychiatry | correction; |
| Indigenously adapted cognitive-behavioral therapy for excessive smartphone use (IACBT-ESU): A randomized controlled trial. | Khalily, Muhammad Tahir; Bhatti, Mujeeb Masud; Ahmad, Irshad; Saleem, Tamkeen; Hallahan, Brian; Ali, Syeda Ayat-e-Zainab; Khan, Ahmad Ali; Hussain, Basharat | 2021 | Psychology of Addictive Behaviors | Non-digital intervention; |
| Online mindfulness stress intervention for family carers of children and adults with intellectual disabilities: Feasibility randomized controlled trial. | Flynn, Samantha; Hastings, Richard P; Burke, Christine; Howes, Simon; Lunsky, Yona; Weiss, Jonathan A; Bailey, Tom | 2020 | Mindfulness | No confirmed mental health or neurodevelopmental diagnosis; |
| Comparison of face-to-face and remote support for interventionists learning to deliver JASPER intervention with children who have autism. | Shire, Stephanie Y; Worthman, Lisa Baker; Shih, Wendy; Kasari, Connie; | 2020 | Journal of Behavioral Education | No confirmed mental health or neurodevelopmental diagnosis; |
| Effectiveness of an Internet-Based and Telephone-Assisted Training for Parents of 4-Year-Old Children With Disruptive Behavior: implementation Research | Sourander, A; Ristkari, T; Kurki, M; Gilbert, S; Hinkka-Yli-SalomÃ¤ki, S; Kinnunen, M; Pulkki-RÃ¥back, L; McGrath, PJ | 2022 |  | No confirmed mental health or neurodevelopmental diagnosis; |
| Effectiveness of telemedicine interventions in children with Autism spectrum disorder: a randomized controlled trial study | TCTR20250117004, | 2025 | N/A | Full text not published/available after librarian support; |
| Delivering cognitive behavior therapy (CBT) for children and adolescents via videoconferencing: a partially randomized patient preferences (PRPP) feasibility study | DRKS00029172, | 2022 | N/A | Full text not published/available after librarian support; |
| SupporTing pARents and their autistic Children through Anxiety Treatment (STAR-CAT) | ISRCTN57543210, | 2025 | N/A | protocol; |
| A prospective, multicenter, randomized controlled clinical trial to evaluate ADHD-assisted therapy software in the treatment of children with attention deficit hyperactivity disorder | ChiCTR2400085638, | 2024 | N/A | Full text not published/available after librarian support; |
| The Effectiveness and Cost-Effectiveness of a Universal Digital Parenting Intervention Designed and Implemented During the COVID-19 Pandemic: evidence From a Rapid-Implementation Randomized Controlled Trial Within a Cohort | Palmer, M; Beckley-Hoelscher, N; Shearer, J; Kostyrka-Allchorne, K; Robertson, O; Koch, M; Pearson, O; Slovak, P; Day, C; Byford, S; et al. | 2023 | JMIR | No confirmed mental health or neurodevelopmental diagnosis; |
| A randomized controlled trial on the effectiveness of internet-delivered parent-child interaction therapy in children with attention-deficit/hyperactivity disorder | UMIN000047166, | 2022 | N/A | Full text not published/available after librarian support; |
| The effect of telerehabilitation sensory integration therapy applied to Autism spectrum disorder (ASD) children on sensory function and parental satisfaction : randomised conrolled trial | KCT0007012, | 2022 | N/A | Full text not published/available after librarian support; |
| Effectiveness of smartphone behavior activation, assertion training, and problem-solving therapy for parenting stress of parents raising children with autism spectrum disorder: multicenter, randomized, open-label, parallel group comparison trial | JPRN-UMIN000052319, | 2023 | N/A | protocol; |
| Effectiveness of a technology-assisted, family volunteers delivered, brief, multicomponent parents' skills training intervention for children with developmental disorders in rural Pakistan: a cluster randomized controlled trial | Hamdani, SU; Zill-e-Huma; Suleman, N; Akhtar, P; Nazir, H; Masood, A; Tariq, M; Koukab, A; Salomone, E; Pacione, L; Brown, F; Shire, S; Sikander, S; Servili, C; Wang, DL; Minhas, FA; Rahman, A | 2021 | International Journal of Mental Health Systems | No confirmed mental health or neurodevelopmental diagnosis; |
| Exposure Therapy in Mixed Reality for Obsessive-Compulsive Disorder: A Randomized Clinical Trial | Miegel F.; Jelinek L.; Lohse L.; Moritz S.; Blomer J.; Juckoff K.; Yassari A.; Rolvien L. | 2025 | JAMA Network Open | Non-digital intervention; |
| Internet and Telephone Support for Discontinuing Long-Term Antidepressants: The REDUCE Cluster Randomized Trial | Kendrick T.; Stuart B.; Bowers H.; Haji Sadeghi M.; Page H.; Dowrick C.; Moore M.; Gabbay M.; Leydon G.M.; Yao G.L.; Little P.; Griffiths G.; Lewis G.; May C.; Moncrieff J.; Johnson C.F.; MacLeod U.; Gilbody S.; Dewar-Haggart R.; Williams S.; O'Brien W.; Tiwari R.; Woods C.; Patel T.; Khan N.; Van Ginneken N.; Din A.; Reidy C.; Lucier R.; Palmer B.; Becque T.; Van Leeuwen E.; Zhu S.; Geraghty A.W.A. | 2024 | JAMA Network Open | Non-digital intervention; |
| A Randomized Controlled Trial of an Online Mindfulness Program for Adolescents at Risk for Internalizing Problems | Garber J.; Chavira D.A.; Adam E.K.; Craske M.G.; McMahon T.; Williams A.; Abitante G.; Lanser I.; Pashtunyar D.S.; Chen S.; Zinbarg R. | 2025 | Journal of Consulting and Clinical Psychology | No confirmed mental health or neurodevelopmental diagnosis; |
| Managing Depression in Rural Depressed Cardiac Patients in the Face of Perceived Depression Stigma: A Randomized Controlled Trial of Two Types of Online Cognitive Behavioral Therapy | Moser D.; Wu J.-R.; Latimer A.; Kang J.H.; Lin C.-Y.; Chung M. | 2024 | Circulation | Abstract-insufficient data; |
| Clinical outcomes of asynchronous telerehabilitation through a mobile app are equivalent to synchronous telerehabilitation in patients with fibromyalgia: a randomized control study | Timurtas E.; Huzmeli I.; Demirbuken I.; Polat M.G. | 2025 | BMC Musculoskeletal Disorders | No confirmed mental health or neurodevelopmental diagnosis; |
| Effectiveness of video-based psychiatric rehabilitation for patients with early-phase schizophrenia spectrum: A randomized controlled trial | Zhou C.; Li H.; Zhang Z.; Li S.; Wu S.; Dai Y.; Zhang X.; Lai H.; Zhou Q.; Yang M.; Zuo X.; Ning J.; Wu J.; Zheng T.; Li N.; Zou X.; Liu L.; Luo X.; Yang J.; Wang Y.; Zheng L.; Luo Y.; Chen Y.; Deng H. | 2025 | Early Intervention in Psychiatry | Non-digital intervention; |
| Effectiveness of a Web-Based Individual Coping and Alcohol Intervention Program for Children of Parents With Alcohol Use Problems: Randomized Controlled Trial | Wall H.; Hansson H.; Zetterlind U.; Kvillemo P.; Elgan T.H. | 2024 | Journal of Medical Internet Research | No confirmed mental health or neurodevelopmental diagnosis; |
| Impact of an Online Discussion Forum on Self-Guided Internet-Delivered Cognitive Behavioral Therapy for Public Safety Personnel: Randomized Trial | McCall H.C.; Hadjistavropoulos H.D. | 2024 | Journal of Medical Internet Research | No confirmed mental health or neurodevelopmental diagnosis; |
| Web-Based Coping Skills Training and Coach Support for Women Living With a Partner With an Alcohol Use Disorder: Randomized Controlled Trial | Rychtarik R.G.; Danaher B.G.; McGillicuddy N.B.; Tyler M.S.; Barrick C.; Leong F.; Kosty D.B. | 2024 | Journal of Medical Internet Research | No confirmed mental health or neurodevelopmental diagnosis; |
| Effectiveness and cost-effectiveness of online recorded recovery narratives in improving quality of life for people with non-psychotic mental health problems: a pragmatic randomized controlled trial | Slade M.; Rennick-Egglestone S.; Elliott R.; Newby C.; Robinson C.; Gavan S.P.; Paterson L.; Ali Y.; Yeo C.; Glover T.; Pollock K.; Callard F.; Priebe S.; Thornicroft G.; Repper J.; Keppens J.; Smuk M.; Franklin D.; Walcott R.; Harrison J.; Smith R.; Robotham D.; Bradstreet S.; Gillard S.; Cuijpers P.; Farkas M.; Zeev D.B.; Davidson L.; Kotera Y.; Roe J.; Ng F.; Llewellyn-Beardsley J. | 2024 | World Psychiatry | No confirmed mental health or neurodevelopmental diagnosis; |
| The Effectiveness of a Digital App for Reduction of Clinical Symptoms in Individuals With Panic Disorder: Randomized Controlled Trial | Kim K.; Hwang H.; Bae S.; Kim S.M.; Han D.H. | 2024 | Journal of Medical Internet Research | No other elements of trial design apart from intervention are online |
| Methodological approaches in developing and implementing digital health interventions amongst underserved women | Crawford A.D.; Slavin R.; Tabar M.; Radhakrishnan K.; Wang M.; Estrada A.; McGrath J.M. | 2024 | Public Health Nursing | No confirmed mental health or neurodevelopmental diagnosis; |
| Layperson-Delivered Telephone-Based Behavioral Activation among Low-Income Older Adults during the COVID-19 Pandemic: The HEAL-HOA Randomized Clinical Trial | Kwok J.Y.Y.; Jiang D.; Yeung D.Y.-L.; Choi N.G.; Ho R.T.H.; Warner L.M.; Chou K.-L. | 2024 | JAMA Network Open | No confirmed mental health or neurodevelopmental diagnosis; |
| Barriers to using eHealth/mHealth platforms and perceived beneficial eHealth/mHealth platform features among informal carers of persons living with dementia: a qualitative study | Samari E.; Yuan Q.; Zhang Y.; Jeyagurunathan A.; Subramaniam M. | 2024 | BMC geriatrics | No confirmed mental health or neurodevelopmental diagnosis; |
| Engagement with a Mobile Chat-Based Intervention for Smoking Cessation: A Secondary Analysis of a Randomized Clinical Trial | Li Y.; Luk T.T.; Cheung Y.T.D.; Zhao S.; Zeng Y.; Tong H.S.C.; Lai V.W.Y.; Wang M.P. | 2024 | JAMA Network Open | No confirmed mental health or neurodevelopmental diagnosis; |
| Participants' experience with an online mindfulness-based stress reduction intervention for anxiety management in breast cancer survivors: A secondary analysis of a randomized trial | Verduzco-Aguirre H.C.; Mansilla M.P.; Salazar-Alejo M.; Ornelas J.G.; Mesa-Chavez F.; Guajardo A.F.; Villarreal-Garza C. | 2024 | Cancer Research | No confirmed mental health or neurodevelopmental diagnosis; |
| Effectiveness of a Digital Health Intervention Leveraging Reinforcement Learning: Results From the Diabetes and Mental Health Adaptive Notification Tracking and Evaluation (DIAMANTE) Randomized Clinical Trial | Aguilera A.; Avalos M.A.; Xu J.; Chakraborty B.; Figueroa C.; Garcia F.; Rosales K.; Hernandez-Ramos R.; Karr C.; Williams J.; Ochoa-Frongia L.; Sarkar U.; Yom-Tov E.; Lyles C. | 2024 | Journal of Medical Internet Research | Not emotional/behavioural related outcomes; |
| Effects of Wearable Fitness Trackers and Activity Adequacy Mindsets on Affect, Behavior, and Health: Longitudinal Randomized Controlled Trial | Zahrt O.H.; Evans K.; Murnane E.; Santoro E.; Baiocchi M.; Landay J.; Delp S.; Crum A. | 2023 | Journal of Medical Internet Research | No confirmed mental health or neurodevelopmental diagnosis; |
| Effectiveness of an Immersive Telemedicine Platform for Delivering Diabetes Medical Group Visits for African American, Black and Hispanic, or Latina Women With Uncontrolled Diabetes: The Women in Control 2.0 Noninferiority Randomized Clinical Trial | Mitchell S.E.; Bragg A.; De La Cruz B.A.; Winter M.R.; Reichert M.J.; Laird L.D.; Moldovan I.A.; Parker K.N.; Martin-Howard J.; Gardiner P. | 2023 | Journal of Medical Internet Research | No confirmed mental health or neurodevelopmental diagnosis; |
| Effectiveness of Digital Guided Self-help Mindfulness Training During Pregnancy on Maternal Psychological Distress and Infant Neuropsychological Development: Randomized Controlled Trial | Zhang X.; Li Y.; Wang J.; Mao F.; Wu L.; Huang Y.; Sun J.; Cao F. | 2023 | Journal of Medical Internet Research | No other elements of trial design apart from intervention are online |
| Differences Between Online Trial Participants Who Have Used Statutory Mental Health Services and Those Who Have Not: Analysis of Baseline Data From 2 Pragmatic Trials of a Digital Health Intervention | Rennick-Egglestone S.; Newby C.; Robinson C.; Yeo C.; Ng F.; Elliott R.A.; Ali Y.; Llewellyn-Beardsley J.; Pomberth S.; Harrison J.; Gavan S.P.; Cuijpers P.; Priebe S.; Hall C.L.; Slade M. | 2023 | Journal of Medical Internet Research | No confirmed mental health or neurodevelopmental diagnosis; |
| Healthy lifestyle promotion via digital self-help for mental health patients in primary care: a pilot study including an embedded randomized recruitment trial | Kolaas K.; Axelsson E.; Hedman-Lagerlof E.; Berman A.H. | 2023 | Primary health care research & development | No confirmed mental health or neurodevelopmental diagnosis; |
| Integrating Text Messaging in a Low Threshold Telebuprenorphine Program for New York City Residents with Opioid Use Disorder during COVID-19: A Pilot Randomized Controlled Trial | Tofighi B.; Badiei B.; Badolato R.; Lewis C.F.; Nunes E.; Thomas A.; Lee J.D. | 2023 | Journal of Addiction Medicine | Not emotional/behavioural related outcomes; |
| Gaming disorder and stigma-related judgements of gaming individuals: An online randomized controlled trial | Galanis C.R.; Weber N.; Delfabbro P.H.; Billieux J.; King D.L. | 2023 | Addiction | No confirmed mental health or neurodevelopmental diagnosis; |
| Effect of an Individually Tailored and Home-Based Intervention in the Chronic Phase of Traumatic Brain Injury: A Randomized Clinical Trial | Borgen I.M.H.; Lovstad M.; Hauger S.L.; Forslund M.V.; Kleffelgard I.; Andelic N.; Sveen U.; Soberg H.L.; Sigurdardottir S.; Winter L.; Lindstad M.O.; Brunborg C.; Roe C. | 2023 | JAMA Network Open | No confirmed mental health or neurodevelopmental diagnosis; |
| A Randomized Clinical Trial Testing the Effect of Digital Behavioral Interventions on Pain and Mental Health Among Adults with Sickle Cell Disease | Jonassaint C.R.; Lalama C.M.; O'Brien J.A.; Badawy S.M.; Hamm M.E.; Stinson J.; Lalloo C.; Carroll C.P.; Saraf S.L.; Gordeuk V.R.; Cronin R.; Shah N.; Lanzkron S.M.; Liles D.K.; Saint-Jean L.; DeBaun M.R.; Trimnell C.; Bailey L.; Lawrence R.H.; Decastro L.M.; Palermo T.; Abebe K. | 2023 | Blood | No confirmed mental health or neurodevelopmental diagnosis; |
| Evaluating REACHOUT-A Mobile App That Delivers PeerLed Mental Health Support to Adults with Type 1 Diabetes | Tang T.S.; Klein G.; Gorges M.; Fisher L.; Polonsky W.H.; Hessler D.M.; Taylor D. | 2023 | Diabetes | Abstract-insufficient data; |
| The wellbeing neuro course: a randomised controlled trial of an internet-delivered transdiagnostic psychological intervention for adults with neurological disorders | Gandy M.; Heriseanu A.I.; Balakumar T.; Karin E.; Walker J.; Hathway T.; Bisby M.A.; Scott A.J.; Dudeney J.; Fisher A.; Titov N.; Dear B.F. | 2023 | Psychological medicine | No confirmed mental health or neurodevelopmental diagnosis; |
| Facilitating the transition to posttreatment survivorship for adolescent and young adults: A randomized waitlist control trial of a virtual mind-body intervention | Perez G.K.; Finkelstein-Fox L.; Markwart M.; Mizrach H.; Recklitis C.J.; Goshe B.M.; Kenney L.B.; Greer J.A.; Miller K.K.; Chang Y.; Peppercorn J.M.; Denninger J.W.; Park E.R. | 2023 | Journal of Clinical Oncology | No confirmed mental health or neurodevelopmental diagnosis; |
| Effect of an eHealth intervention on older adults' quality of life and health-related outcomes: a randomized clinical trial | Kornfield R.; Mares M.-L.; Johnston D.C.; Cody O.J.; Yang E.F.; Gustafson D.H.; Hwang J.; Mahoney J.E.; Curtin J.J.; Tahk A.; Shah D.V. | 2022 | Journal of General Internal Medicine | No confirmed mental health or neurodevelopmental diagnosis; |
| Durability of the Treatment Effects of an 8-Week Self-administered Home-Based Virtual Reality Program for Chronic Low Back Pain: 6-Month Follow-up Study of a Randomized Clinical Trial | Garcia L.; Birckhead B.; Krishnamurthy P.; Mackey I.; Sackman J.; Salmasi V.; Louis R.; Castro C.; Maddox R.; Maddox T.; Darnall B.D. | 2022 | Journal of Medical Internet Research | No confirmed mental health or neurodevelopmental diagnosis; |
| Guided digital health intervention for depression in Lebanon: randomised trial | Cuijpers P.; Heim E.; Ramia J.A.; Burchert S.; Carswell K.; Cornelisz I.; Knaevelsrud C.; Noun P.; van Klaveren C.; Van't Hof E.; Zoghbi E.; van Ommeren M.; El Chammay R. | 2022 | Evidence-based mental health | No confirmed mental health or neurodevelopmental diagnosis; |
| The Effect of M-Health-Based Core Stability Exercise Combined with Self-Compassion Training for Patients with Nonspecific Chronic Low Back Pain: A Randomized Controlled Pilot Study | Zheng F.; Zheng Y.; Liu S.; Yang J.; Xiao W.; Chen L.; Yang W.; Zhang S.; Yu Q.; Hao Z.; Wang Y.; Wang C. | 2022 | Pain and Therapy | No confirmed mental health or neurodevelopmental diagnosis; |
| An online intervention for 18-25-year-old youth whose parents have a mental illness and/or substance use disorder: A pilot randomized controlled trial | Maybery D.; Reupert A.; Bartholomew C.; Cuff R.; Duncan Z.; McAuliffe C.; McLean L.; Pettenuzzo L.; Swing A.; Foster K. | 2022 | Early Intervention in Psychiatry | No confirmed mental health or neurodevelopmental diagnosis; |
| Challenges and opportunities in conducting research with older adults with dementia during COVID-19 and beyond | Sharma R.K.; Teng A.; Asirot M.G.; Taylor J.O.; Borson S.; Turner A.M. | 2022 | Journal of the American Geriatrics Society | Not RCT (or based on trial); |
| Internet-Based CBT for Somatic Symptom Distress (iSOMA) in Emerging Adults: A Randomized Controlled Trial | Hennemann S.; Bohme K.; Kleinstauber M.; Baumeister H.; Kuchler A.-M.; Ebert D.D.; Witthoft M. | 2022 | Journal of Consulting and Clinical Psychology | No confirmed mental health or neurodevelopmental diagnosis; |
| Clinical Predictors of Engagement in Teleintegrated Care and Telereferral Care for Complex Psychiatric Disorders in Primary Care: a Randomized Trial | Severe J.; Pfeiffer P.N.; Palm-Cruz K.; Hoeft T.; Sripada R.; Hawrilenko M.; Chen S.; Fortney J. | 2022 | Journal of General Internal Medicine | Not emotional/behavioural related outcomes; |
| Technology-enabled collaborative care for youth with early psychosis: Results of a feasibility study to improve physical health behaviours | Melamed O.; Voineskos A.; Vojtila L.; Ashfaq I.; Veldhuizen S.; Dragonetti R.; Carriere R.; LaChance L.; Kohut S.A.; Tulloch T.; Argarwal S.M.; Hahn M.; Mulsant B.H.; Selby P. | 2022 | Early Intervention in Psychiatry | Not emotional/behavioural related outcomes; |
| Effects of an Explicit Value Clarification Method With Computer-Tailored Advice on the Effectiveness of a Web-Based Smoking Cessation Decision Aid: Findings From a Randomized Controlled Trial | Gultzow T.; Smit E.S.; Crutzen R.; Jolani S.; Hoving C.; Dirksen C.D. | 2022 | Journal of Medical Internet Research | No confirmed mental health or neurodevelopmental diagnosis; |
| Effects of a Smartphone-Based Out-of-Hospital Screening App for Neonatal Hyperbilirubinemia on Neonatal Readmission Rates and Maternal Anxiety: Randomized Controlled Trial | Yan Q.; Gong Y.; Luo Q.; Yin X.; Yang L.; Wang H.; Feng J.; Xing K.; Huang Y.; Huang C.; Fan L. | 2022 | Journal of Medical Internet Research | No confirmed mental health or neurodevelopmental diagnosis; |
| The Brain Health Champion Study: A Health Coaching Intervention with Mobile Technology in Older Adults with Mild Cognitive Impairment or Risk Factorsfor Dementia | Salazar S.R.; Krivanek T.; McFeeley B.; Nicastri C.; Babazadeh D.; Daffner K.; Gale S. | 2022 | Neurology | Abstract-insufficient data; |
| AN ONLINE SELF-DIRECTED PROGRAM FOR INDIVIDUALS SHARING A CHILD WITH A CO-PARENT WITH PROBLEMATIC ALCOHOL CONSUMPTION: A RANDOMIZED | Siljeholm O.; Lindner P.; Johansson M.; Hammarberg A. | 2022 | Alcoholism: Clinical and Experimental Research | Abstract-insufficient data; |
| A brief intervention to increase uptake and adherence of an internet-based program for depression and anxiety (Enhancing Engagement with Psychosocial Interventions): Randomized controlled trial | Batterham P.J.; Calear A.L.; Sunderland M.; Kay-Lambkin F.; Farrer L.M.; Christensen H.; Gulliver A. | 2021 | Journal of Medical Internet Research | No confirmed mental health or neurodevelopmental diagnosis; |
| A Telehealth-supported, Integrated care with CHWs, and MEdication-access (TIME) Program for Diabetes Improves HbA1c: a Randomized Clinical Trial | Vaughan E.M.; Hyman D.J.; Naik A.D.; Samson S.L.; Razjouyan J.; Foreyt J.P. | 2021 | Journal of General Internal Medicine | No confirmed mental health or neurodevelopmental diagnosis; |
| Effect of a Telecare Case Management Program for Older Adults Who Are Homebound during the COVID-19 Pandemic: A Pilot Randomized Clinical Trial | Wong A.K.C.; Wong F.K.Y.; Chow K.K.S.; Wong S.M.; Lee P.H. | 2021 | JAMA Network Open | No confirmed mental health or neurodevelopmental diagnosis; |
| Virtual reality cognitive training among individuals with alcohol use disorder undergoing residential treatment: Pilot randomized controlled trial | Gamito P.; Oliveira J.; Matias M.; Cunha E.; Brito R.; Lopes P.F.; Deus A. | 2021 | Journal of Medical Internet Research | No other elements of trial design apart from intervention are online; |
| Project Khanya: results from a pilot randomized type 1 hybrid effectiveness-implementation trial of a peer-delivered behavioural intervention for ART adherence and substance use in HIV care in South Africa | Magidson J.F.; Joska J.A.; Belus J.M.; Andersen L.S.; Regenauer K.S.; Rose A.L.; Myers B.; Majokweni S.; O'Cleirigh C.; Safren S.A. | 2021 | Journal of the International AIDS Society | No confirmed mental health or neurodevelopmental diagnosis; |
| A smartphone intervention for people with serious mental illness: Fully remote randomized controlled trial of CORE | Ben-Zeev D.; Chander A.; Tauscher J.; Buck B.; Nepal S.; Campbell A.; Doron G. | 2021 | Journal of Medical Internet Research | No confirmed mental health or neurodevelopmental diagnosis; |
| The effectiveness of a web-based self-help program to reduce alcohol use among adults with drinking patterns considered harmful, hazardous, or suggestive of dependence in four low-and middle-income countries: Randomized controlled trial | Schaub M.P.; Tiburcio M.; Martinez-Velez N.; Ambekar A.; Bhad R.; Wenger A.; Baumgartner C.; Padruchny D.; Osipchik S.; Poznyak V.; Rekve D.; Moraes F.L.; Monezi Andrade A.L.; Oliveira Souza-Formigoni M.L. | 2021 | Journal of Medical Internet Research | No confirmed mental health or neurodevelopmental diagnosis; |
| The baby steps web program for the well-being of new parents: Randomized controlled trial | Kavanagh D.J.; Connolly J.; Fisher J.; Kim Halford W.; Hamilton K.; Hides L.; Milgrom J.; Rowe H.; Scuffham P.A.; White K.M.; Wittkowski A.; Appleton S.; Sanders D. | 2021 | Journal of Medical Internet Research | No confirmed mental health or neurodevelopmental diagnosis; |
| Effect of a WeChat-Based Intervention (Run4Love) on Depressive Symptoms Among People Living With HIV in China: A Randomized Controlled Trial | Guo Y.; Hong Y.A.; Cai W.; Li L.; Hao Y.; Qiao J.; Xu Z.; Zhang H.; Zeng C.; Liu C.; Li Y.; Zhu M.; Zeng Y.; Penedo F.J. | 2020 | Journal of medical Internet research | No other elements of trial design apart from intervention are online; |
| A randomized clinical trial of mobile phone motivational interviewing for alcohol use problems in Kenya | Harder V.S.; Musau A.M.; Musyimi C.W.; Ndetei D.M.; Mutiso V.N. | 2020 | Addiction | Before 2020; |
| Feasibility and effectiveness of a telehealth service delivery model for treating childhood posttraumatic stress: A community-based, open pilot trial of trauma-focused cognitive-behavioral therapy | Stewart R.W.; Orengo-Aguayo R.; Young J.; Wallace M.M.; Cohen J.A.; Mannarino A.P.; de Arellano M.A. | 2020 | Journal of Psychotherapy Integration | Not RCT (or based on trial); |
| Effect of a Videoconference-Based Online Group Intervention for Traumatic Stress in Parents of Children with Life-threatening Illness: A Randomized Clinical Trial | Muscara F.; McCarthy M.C.; Rayner M.; Nicholson J.M.; Dimovski A.; McMillan L.; Hearps S.J.C.; Yamada J.; Burke K.; Walser R.; Anderson V.A. | 2020 | JAMA Network Open | No confirmed mental health or neurodevelopmental diagnosis; |
| A randomised trial comparing a brief online delivery of mindfulness-plus-values versus values only for symptoms of depression: Does baseline severity matter? | Kingston J.; Becker L.; Woeginger J.; Ellett L. | 2020 | Journal of Affective Disorders | No confirmed mental health or neurodevelopmental diagnosis; |
| Effect of Telehealth Treatment by Lay Counselors vs by Clinicians on Depressive Symptoms among Older Adults Who Are Homebound: A Randomized Clinical Trial | Choi N.G.; Marti C.N.; Wilson N.L.; Chen G.J.; Sirrianni L.; Hegel M.T.; Bruce M.L.; Kunik M.E. | 2020 | JAMA Network Open | No other elements of trial design apart from intervention are online; |
| Associations Between Global Mental Health and Response to an App-Based Meditation Intervention in Myeloproliferative Neoplasm Patients | Puzia M.E.; Huberty J.; Eckert R.; Larkey L.; Mesa R. | 2020 | Integrative Cancer Therapies | No confirmed mental health or neurodevelopmental diagnosis; |
| Protecting the Mental Health of Small-to-Medium Enterprise Owners: A Randomized Control Trial Evaluating a Self-Administered Versus Telephone Supported Intervention | Martin A.; Kilpatrick M.; Scott J.; Cocker F.; Dawkins S.; Brough P.; Sanderson K. | 2020 | Journal of Occupational and Environmental Medicine | No confirmed mental health or neurodevelopmental diagnosis; |
| Predictors for the Early Termination of a Psychological Intervention during Treatment with Assisted Reproductive Technologies | Bernd M.; Schick M.; Rosner S.; Germeyer A.; Strowitzki T.; Moessner M.; Bauer S.; Ditzen B.; Wischmann T. | 2020 | Geburtshilfe und Frauenheilkunde | No confirmed mental health or neurodevelopmental diagnosis; |
| Telemedicine Cognitive Behavioral Therapy for Anxiety after Stroke: Proof-of-Concept Randomized Controlled Trial | Chun H.-Y.Y.; Carson A.J.; Tsanas A.; Dennis M.S.; Mead G.E.; Calabria C.; Whiteley W.N. | 2020 | Stroke | No confirmed mental health or neurodevelopmental diagnosis; |
| Acceptability and Feasibility of A Digital Health Intervention for Posttraumatic Stress Symptoms: An Entirely Remote Study | Tripp P.; Smirnova M.; Niles A.; Woolley J.; Neylan T.; O'Donovan A. | 2020 | Psychosomatic Medicine | Abstract-insufficient data; |
| Cognitive Training via a Mobile Application to Reduce Obsessive-Compulsive-Related Distress and Cognitions During the COVID-19 Outbreaks: A Randomized Controlled Trial Using a Subclinical Cohort. | Akin-Sari B; Inozu M; Haciomeroglu AB; Cekci BC; Uzumcu E; Doron G | 2022 | Behavioural Therapies | No confirmed mental health or neurodevelopmental diagnosis; |
| A Mobile-Based Intervention to Increase Self-esteem in Students With Depressive Symptoms: Randomized Controlled Trial. | Bruhns A; LÃ¼dtke T; Moritz S; BÃ¼cker L | 2021 | JMIR mental health | No confirmed mental health or neurodevelopmental diagnosis; |
| The Efficacy of "Foundations," a Digital Mental Health App to Improve Mental Well-being During COVID-19: Proof-of-Principle Randomized Controlled Trial. | Catuara-Solarz S; Skorulski B; Estella-Aguerri I; Avella-Garcia CB; Shepherd S; Stott E; Hemmings NR; Ruiz de Villa A; Schulze L; Dix S | 2022 | JMIR mental health | No confirmed mental health or neurodevelopmental diagnosis; |
| Reaching reliable change using short, daily, cognitive training exercises delivered on a mobile application: The case of Relationship Obsessive Compulsive Disorder (ROCD) symptoms and cognitions in a subclinical cohort. | Cerea S; Ghisi M; Bottesi G; Carraro E; Broggio D; Doron G | 2020 | Journal of Affective Disorders | No confirmed mental health or neurodevelopmental diagnosis; |
| Exploring the features of an app-based just-in-time intervention for depression. | Everitt N; Broadbent J; Richardson B; Smyth JM; Heron K; Teague S; Fuller-Tyszkiewicz M | 2021 | Journal of Affective Disorders | No confirmed mental health or neurodevelopmental diagnosis; |
| Multicentre, England-wide randomised controlled trial of the 'Foundations' smartphone application in improving mental health and well-being in a healthcare worker population. | Gnanapragasam SN; Tinch-Taylor R; Scott HR; Hegarty S; Souliou E; Bhundia R; Lamb D; Weston D; Greenberg N; Madan I; Stevelink S; Raine R; Carter B; Wessely S | 2023 | British Journal of Psychiatry | No confirmed mental health or neurodevelopmental diagnosis; |
| Testing the Pragmatic Effectiveness of a Consumer-Based Mindfulness Mobile App in the Workplace: Randomized Controlled Trial. | Huberty JL; Espel-Huynh HM; Neher TL; Puzia ME | 2022 | JMIR Mhealth | No confirmed mental health or neurodevelopmental diagnosis; |
| Evaluation of the Effectiveness of Mobile App-Based Stress-Management Program: A Randomized Controlled Trial. | Hwang WJ; Jo HH | 2019 | International Journal of Environmental Research and Public Health | No confirmed mental health or neurodevelopmental diagnosis; |
| Pilot evaluation of the stop, breathe & think mindfulness app for student clients on a college counseling center waitlist. | Levin ME; Hicks ET; Krafft J | 2022 | Journal of American College Health | No confirmed mental health or neurodevelopmental diagnosis; |
| A randomized controlled trial of a smartphone-based application for the treatment of anxiety. | Newman MG; Jacobson NC; Rackoff GN; Bell MJ; Taylor CB | 2021 | Psychotherapy Research | No confirmed mental health or neurodevelopmental diagnosis; |
| Mindfulness-based mobile app reduces anxiety and increases self-compassion in healthcare students: A randomised controlled trial. | Orosa-Duarte Ã; Mediavilla R; MuÃ±oz-Sanjose A; Palao Ã; Garde J; LÃ³pez-Herrero V; Bravo-Ortiz MF; BayÃ³n C; RodrÃ­guez-Vega B | 2021 | Med Teach | No confirmed mental health or neurodevelopmental diagnosis; |
| A randomised controlled trial of a relationship-focussed mobile phone application for improving adolescents' mental health. | O'Dea B; Han J; Batterham PJ; Achilles MR; Calear AL; Werner-Seidler A; Parker B; Shand F; Christensen H | 2020 | Journal of Child Psychology and Psychiatry | No confirmed mental health or neurodevelopmental diagnosis; |
| Well-being app to support young people during the COVID-19 pandemic: randomised controlled trial. | Thabrew H; Boggiss AL; Lim D; Schache K; Morunga E; Cao N; Cavadino A; Serlachius AS | 2022 | BMJ Open | No confirmed mental health or neurodevelopmental diagnosis; |
| The Impact of a Mindfulness App on Postnatal Distress. | Bear KA; Barber CC; Medvedev ON | 2022 | Mindfulness | No confirmed mental health or neurodevelopmental diagnosis; |
| Trial of a patient-directed eHealth program to ameliorate perinatal depression: the MomMoodBooster2 practical effectiveness study. | Danaher BG; Seeley JR; Silver RK; Tyler MS; Kim JJ; La Porte LM; Cleveland E; Smith DR; Milgrom J; Gau JM | 2023 | American Journal of Obstetrics and Gynaecology | No confirmed mental health or neurodevelopmental diagnosis; |
| Effectiveness of the "Essential Coaching for Every Mother" postpartum text message program on maternal psychosocial outcomes: A randomized controlled trial. | Dol J; Aston M; Grant A; McMillan D; Tomblin Murphy G; Campbell-Yeo M | 2022 | Digital Health | No confirmed mental health or neurodevelopmental diagnosis; |
| A Web-Based, Mindful, and Compassionate Parenting Training for Mothers Experiencing Parenting Stress: Results from a Pilot Randomized Controlled Trial of the Mindful Moment Program. | Fernandes DV; Monteiro F; Canavarro MC; Moreira H | 2022 | Mindfulness | No confirmed mental health or neurodevelopmental diagnosis; |
| A creative and practical approach to postpartum discharge education: Pecha Kucha training via smart phone. | GÃ¼n KakaÅŸÃ§Ä± Ã‡; Durmaz A | 2022 | Health Care Women International | No confirmed mental health or neurodevelopmental diagnosis; |
| Positive intervention effect of mobile health application based on mindfulness and social support theory on postpartum depression symptoms of puerperae. | Liu C; Chen H; Zhou F; Long Q; Wu K; Lo LM; Hung TH; Liu CY; Chiou WK | 2022 | BMC Womens Health | No confirmed mental health or neurodevelopmental diagnosis; |
| The preventive effect of internet-based cognitive behavioral therapy for prevention of depression during pregnancy and in the postpartum period (iPDP): a large scale randomized controlled trial. | Nishi D; Imamura K; Watanabe K; Obikane E; Sasaki N; Yasuma N; Sekiya Y; Matsuyama Y; Kawakami N | 2022 | Psychiatry and Clinical Neurosciences | No confirmed mental health or neurodevelopmental diagnosis; |
| Effect of internet-based cognitive behaviour therapy among women with negative birth experiences on mental health and quality of life - a randomized controlled trial. | SjÃ¶mark J; Svanberg AS; Larsson M; Viirman F; Poromaa IS; Skalkidou A; Jonsson M; Parling T | 2022 | BMC Pregnancy Childbirth | No confirmed mental health or neurodevelopmental diagnosis; |
| Effectiveness of an unguided modular online intervention for highly anxious parents in preventing anxiety in their children: a parallel group randomised controlled trial. | Dunn A; Alvarez J; Arbon A; Bremner S; Elsby-Pearson C; Emsley R; Jones C; Lawrence P; Lester KJ; Morson N; Simner J; Thomson A; Cartwright-Hatton S | 2024 | Lancet Regional Health Europe | No confirmed mental health or neurodevelopmental diagnosis; |
| A novel digital intervention for actively reducing severity of paediatric ADHD (STARS-ADHD): a randomised controlled trial. | Kollins SH; DeLoss DJ; CaÃ±adas E; Lutz J; Findling RL; Keefe RSE; Epstein JN; Cutler AJ; Faraone SV | 2020 | Lancet Digital Health | Non-digital intervention; |
| Gamification as an approach to improve resilience and reduce attrition in mobile mental health interventions: A randomized controlled trial. | Litvin S; Saunders R; Maier MA; LÃttke S | 2020 | PLoS One | No confirmed mental health or neurodevelopmental diagnosis; |
| The Efficacy of Computerized Cognitive Behavioral Therapy for Depressive and Anxiety Symptoms in Patients With COVID-19: Randomized Controlled Trial. | Liu Z; Qiao D; Xu Y; Zhao W; Yang Y; Wen D; Li X; Nie X; Dong Y; Tang S; Jiang Y; Wang Y; Zhao J | 2021 | JMIR | Non-digital intervention; |
| A Digital Human for Delivering a Remote Loneliness and Stress Intervention to At-Risk Younger and Older Adults During the COVID-19 Pandemic: Randomized Pilot Trial. | Loveys K; Sagar M; Pickering I; Broadbent E | 2021 | JMIR Mental Health | No confirmed mental health or neurodevelopmental diagnosis; |
| Feasibility and efficacy of a digital CBT intervention for symptoms of Generalized Anxiety Disorder: A randomized multiple-baseline study. | Miller CB; Gu J; Henry AL; Davis ML; Espie CA; Stott R; Heinz AJ; Bentley KH; Goodwin GM; Gorman BS; Craske MG; Carl JR | 2021 | Journal of Behaviour Therapy and Experimental Psychiatry | Not RCT (or based on trial); |
| A randomized controlled trial of a digital cognitive-behavioral therapy program (COMPASS) for managing depression and anxiety related to living with a long-term physical health condition. | Picariello F; Hulme K; Seaton N; Hudson JL; Norton S; Wroe A; Moss-Morris R | 2024 | Psychological Medicine | No confirmed mental health or neurodevelopmental diagnosis; |
| A pilot randomized controlled trial of a group intervention via Zoom to relieve loneliness and depressive symptoms among older persons during the COVID-19 outbreak. | Shapira S; Yeshua-Katz D; Cohn-Schwartz E; Aharonson-Daniel L; Sarid O; Clarfield AM | 2021 | Internet Interventions | No confirmed mental health or neurodevelopmental diagnosis; |
| Single-session digital intervention for adolescent depression, anxiety, and well-being: Outcomes of a randomized controlled trial with Kenyan adolescents. | Osborn TL; Rodriguez M; Wasil AR; Venturo-Conerly KE; Gan J; Alemu RG; Roe E; Arango G S; Otieno BH; Wasanga CM; Shingleton R; Weisz JR | 2020 | Journal of Consulting and Clinical Psychology | Non-digital intervention; |
| A comparison of virtual reality and verbal imaginal exposure for childhood anxiety disorders. | Biggs BK; Whiteside SPH; Knutson M; Seifert S; Hofschulte DR; Geske JR; Lass AN; Vickers KS; Gloe LM; Reneson-Feeder ST | 2025 | Journal of Anxiety Disorders | Non-digital intervention; |
| Training rapid automatized naming in children with developmental Dyslexia. | Graziani D; Capodieci A; Casalini C; Giaccherini S; Scali V; Luccherino L; Pecini C | 2025 | Child Neuropsychology | Not emotional/behavioural related outcomes; |
| Effectiveness of a Virtual Reality Serious Video Game (The Secret Trail of Moon) for Emotional Regulation in Children With Attention-Deficit/Hyperactivity Disorder: Randomized Clinical Trial. | Martin-Moratinos M; Bella-FernÃ¡ndez M; Rodrigo-Yanguas M; GonzÃ¡lez-TardÃ³n C; Li C; Wang P; Royuela A; Lopez-Garcia P; Blasco-Fontecilla H | 2025 | JMIR Serious Games | Non-digital intervention; |
| A 5-week Digital Intervention to Reduce Attention Problems in Children With ADHD: A Double-Blind Randomized Controlled Trial. | Kirk HE; Richmond S; Gaunson T; Bennett M; Herschtal A; Bellgrove M; Cornish K | 2024 | Journal of Attention Disorders | No other elements of trial design apart from intervention are online; |
| Behavioral skill practice as a predictor of mood and family functioning in adolescents with bipolar and depressive mood disorders: Results of a 6-month randomized trial of family-focused therapy. | Weintraub MJ; Merranko JA; Ichinose MC; Denenny DM; Walshaw PD; Morgan-Fleming G; Brown RD; Arevian AC; Miklowitz DJ | 2024 | Bipolar Disorders | Change in practice due to COVID |
| Telehealth coaching in Project ImPACT indirectly affects children's expressive language ability through parent intervention strategy use and child intentional communication: An RCT. | Ingersoll B; Frost KM; Straiton D; Ramos AP; Casagrande K | 2024 | Autism Researc | No other elements of trial design apart from intervention are online; |
| A Randomized Trial of the Accuracy of Novel Telehealth Instruments for the Assessment of Autism in Toddlers. | Corona LL; Wagner L; Hooper M; Weitlauf A; Foster TE; Hine J; Miceli A; Nicholson A; Stone C; Vehorn A; Warren Z | 2024 | Journal of Autism and Developmental Disorders | No confirmed mental health or neurodevelopmental diagnosis; |
| A Digital Cognitive-Physical Intervention for Attention-Deficit/Hyperactivity Disorder: Randomized Controlled Trial. | Zhao L; Agazzi H; Du Y; Meng H; Maku R; Li K; Aspinall P; Garvan CW; Fang S | 2024 | J Medical Internet Research | Non-digital intervention; |
| A Randomized Controlled Trial Comparing Face-to-Face Versus Remote Delivery of Low-Tech Augmentative and Alternative Communication in Nonspeaking Children With Autism Spectrum Disorder. | Mishra A | 2024 | Journal of Speech Language and Hearing Research | No other elements of trial design apart from intervention are online; |
| Effects of a Nonwearable Digital Therapeutic Intervention on Preschoolers With Autism Spectrum Disorder in China: Open-Label Randomized Controlled Trial. | Chu L; Shen L; Ma C; Chen J; Tian Y; Zhang C; Gong Z; Li M; Wang C; Pan L; Zhu P; Wu D; Wang Y; Yu G | 2023 | Journal of Medical Internet Research | Non-digital intervention; |
| An Evaluation of a Mobile Application Designed to Teach Receptive Language Skills to Children with Autism Spectrum Disorder. | Novack MN; Hong E; Dixon DR; Granpeesheh D | 2019 | Behaviour Analysis in Practice | Before 2020; |
| Effectiveness of IMPUTE ADT-1 mobile application in children with autism spectrum disorder: An interim analysis of an ongoing randomized controlled trial. | Panda PK; Elwadhi A; Gupta D; Palayullakandi A; Tomar A; Singh M; Vyas A; Kumar D; Sharawat IK | 2024 | Journal of Neurosciences in Rural Practice | No other elements of trial design apart from intervention are online; |
| A Randomized Clinical Trial of Technology-Enhanced Family-Focused Therapy for Youth in the Early Stages of Mood Disorders. | Miklowitz DJ; Weintraub MJ; Ichinose MC; Denenny DM; Walshaw PD; Wilkerson CA; Frey SJ; Morgan-Fleming GM; Brown RD; Merranko JA; Arevian AC | 2023 | JAACAP Open | Change in practice due to COVID |
| How does therapist guided game-based intervention program effect motor skills in children with Attention Deficit Hyperactivity Disorder?: Single blind randomised study design. | BarkÄ±n K; Ege T; Ã–zgÃ¼n KK; Koray K; Sedef Åž | 2023 | Research in Developmental Disabilities | No other elements of trial design apart from intervention are online |
| Virtual reality technology enhances the cognitive and social communication of children with autism spectrum disorder. | Zhao J; Zhang X; Lu Y; Wu X; Zhou F; Yang S; Wang L; Fei F | 2022 | Frontiers in Public Health | Non-digital intervention; |
| Comparing the Effect of Risperidone, Virtual Reality and Risperidone on Social Skills, and Behavioral Problems in Children with Autism: A Follow-up Randomized Clinical Trial. | Soltani Kouhbanani S; Khosrorad R; Zarenezhad S; Arabi SM | 2021 | Archives of Iranian Medicine | No other elements of trial design apart from intervention are online |
| Virtual Remediation Versus Methylphenidate to Improve Distractibility in Children With ADHD: A Controlled Randomized Clinical Trial Study. | Bioulac S; Micoulaud-Franchi JA; Maire J; Bouvard MP; Rizzo AA; Sagaspe P; Philip P | 2020 | Journal of Attention Disorders | Non-digital intervention; |
| A Randomised Controlled Trial of an Information Communication Technology Delivered Intervention for Children with Autism Spectrum Disorder Living in Regional Australia. | Parsons D; Cordier R; Lee H; Falkmer T; Vaz S | 2019 | Journal of Autism and Developmental Disorders | Before 2020; |
| Efficacy and cost-effectiveness of therapist-guided internet cognitive behavioural therapy for paediatric anxiety disorders: a single-centre, single-blind, randomised controlled trial. | Jolstedt M; Wahlund T; Lenhard F; LjÃ³tsson B; Mataix-Cols D; Nord M; Ã–st LG; HÃ¶gstrÃ¶m J; Serlachius E; Vigerland S | 2018 | Lancet Child Adolescent Health | Before 2020; |
| Remote Collaborative Depression Care Program for Adolescents in AraucanÃ­a Region, Chile: Randomized Controlled Trial. | MartÃ­nez V; Rojas G; MartÃ­nez P; Zitko P; IrarrÃ¡zaval M; Luttges C; Araya R | 2018 | Journal of Medical Internet Research | Non-digital intervention; |
| Learning better by repetition or variation? Is transfer at odds with task specific training? | Bonney E; Jelsma LD; Ferguson GD; Smits-Engelsman BC | 2017 | PLoS One | Before 2020; |
| A randomized controlled trial of internet-delivered cognitive behaviour therapy for adolescent anxiety disorders in a routine clinical care setting with and without parent sessions. | Waite P; Marshall T; Creswell C | 2019 | Child Adolescent Mental Health | Before 2020; |
| Lidcombe Program Webcam Treatment for Early Stuttering: A Randomized Controlled Trial. | Bridgman K; Onslow M; O'Brian S; Jones M; Block S | 2016 | Journal of Speech Language and Hearing Research | Before 2020; |
| A RANDOMIZED CONTROLLED TRIAL IN COMMUNITY MENTAL HEALTH CENTERS OF COMPUTER-ASSISTED COGNITIVE BEHAVIORAL THERAPY VERSUS TREATMENT AS USUAL FOR CHILDREN WITH ANXIETY. | Storch EA; Salloum A; King MA; Crawford EA; Andel R; McBride NM; Lewin AB | 2015 | Depression and Anxiety | Before 2020; |
| Teletherapy delivery of caregiver behavior training for children with attention-deficit hyperactivity disorder. | Tse YJ; McCarty CA; Stoep AV; Myers KM | 2015 | Telemedicine and E-Health | Before 2020; |
| An initial trial of OPT-In-Early: An online training program for caregivers of autistic children. | Dai YG; Thomas RP; Brennan L; Luu ML; Hughes-Lika J; Reilly M; Moreno P; Obe B; Ahmed KB; Berry LN; Goin-Kochel RP; Helt MS; Barton ML; Dumont-Mathieu T; Robins DL; Fein DA | 2023 | Autism | No other elements of trial design apart from intervention are online |
| Impact of a Web-Portal Intervention on Community ADHD Care and Outcomes. | Epstein JN; Kelleher KJ; Baum R; Brinkman WB; Peugh J; Gardner W; Lichtenstein P; Langberg JM | 2016 | Pediatrics | Before 2020; |
| Behavioral Outcome Effects of Serious Gaming as an Adjunct to Treatment for Children With Attention-Deficit/Hyperactivity Disorder: A Randomized Controlled Trial. | Bul KC; Kato PM; Van der Oord S; Danckaerts M; Vreeke LJ; Willems A; van Oers HJ; Van Den Heuvel R; Birnie D; Van Amelsvoort TA; Franken IH; Maras A | 2016 | Journal of Medical Internet Research | Before 2020; |
| Effectiveness of a telehealth service delivery model for treating attention-deficit/hyperactivity disorder: a community-based randomized controlled trial. | Myers K; Vander Stoep A; Zhou C; McCarty CA; Katon W | 2015 | Journal of the American Academy of Child and Adolescent Psychiatry | Before 2020; |
| A randomized controlled study of remote computerized cognitive, neurofeedback, and combined training in the treatment of children with attention-deficit/hyperactivity disorder. | Luo X; Guo X; Zhao Q; Zhu Y; Chen Y; Zhang D; Jiang H; Wang Y; Johnstone S; Sun L | 2023 | European Child and Adolescent Psychiatry | No other elements of trial design apart from intervention are online |
| Use of Virtual Reality in Children with Dyslexia. | Maresca G; Leonardi S; De Cola MC; Giliberto S; Di Cara M; Corallo F; Quartarone A; PidalÃ  A | 2022 | Children | Non-digital intervention; |
| Effects of Augmented Reality Game-Based Cognitive-Motor Training on Restricted and Repetitive Behaviors and Executive Function in Patients with Autism Spectrum Disorder. | Nekar DM; Lee DY; Hong JH; Kim JS; Kim SG; Seo YG; Yu JH | 2022 | Healthcare | Non-digital intervention; |
| Telehealth Treatment of Behavior Problems in Young Children With Developmental Delay: A Randomized Clinical Trial. | Bagner DM; Berkovits MD; Coxe S; Frech N; Garcia D; Golik A; Heflin BH; Heymann P; Javadi N; Sanchez AL; Wilson MK; Comer JS | 2023 | JAMA Paediatrics | No other elements of trial design apart from intervention are online |
| Effectiveness of a Personalized, Chess-Based Training Serious Video Game in the Treatment of Adolescents and Young Adults With Attention-Deficit/Hyperactivity Disorder: Randomized Controlled Trial. | Rodrigo-Yanguas M; MartÃ­n-Moratinos M; GonzÃ¡lez-TardÃ³n C; Sanchez-Sanchez F; Royuela A; Bella-FernÃ¡ndez M; Blasco-Fontecilla H | 2023 | JMIR Serious Games | No other elements of trial design apart from intervention are online |
| A trial of an iPadâ„¢ intervention targeting social communication skills in children with autism. | Fletcher-Watson S; Petrou A; Scott-Barrett J; Dicks P; Graham C; O'Hare A; Pain H; McConachie H | 2016 | Autism | Before 2020 |
| The impact of a computerised test of attention and activity (QbTest) on diagnostic decision-making in children and young people with suspected attention deficit hyperactivity disorder: single-blind randomised controlled trial. | Hollis C; Hall CL; Guo B; James M; Boadu J; Groom MJ; Brown N; Kaylor-Hughes C; Moldavsky M; Valentine AZ; Walker GM; Daley D; Sayal K; Morriss R | 2018 | Journal of Child Psychology and Psychiatry | Before 2020 |
| Attention and executive functions computer training for attention-deficit/hyperactivity disorder (ADHD): results from a randomized, controlled trial. | Bikic A, Leckman JF, Christensen T, Bilenberg N, Dalsgaard S. | 2018 | Europen Child and Adolescent Psychiatry | Before 2020 |
