# Supplementary material for: Who gets included? Equity in digital and decentralised mental health and neurodevelopmental trials: A systematic review
Source: PLOS Digit Health. 2026 Jun 8;5(6):e0001466. doi: 10.1371/journal.pdig.0001466 (PMC13245764; doi:10.1371/journal.pdig.0001466)
Supplement: S4 Table — S4a. Sociodemographic characteristics as reported by included studies. S4b Table. Sources used to determine sociodemographic characteristics of the country of study*. (DOCX) [file pdig.0001466.s006.docx]

| S4 Table. Sociodemographic characteristics reported (4a) and key sources used (4b)  S4a. Sociodemographic characteristics as reported by included studies | | | | | | | | |  |
| --- | --- | --- | --- | --- | --- | --- | --- | --- | --- |
| Author/Year | **Place** | **Ethnicity** | **Occupation** | **Gender** | **Religion** | **Education** | **Socioeconomic status** | **Social capital** | **Age** |
| Andrews et al. (2023) | Rural (n = 75; 72.8%); urban (n = 28; 27.2%) | Not reported | Employed (n = 58; 56.3%); not employed (n = 33; 32.1%) | Female (n = 66; 64.1%); male (n = 34; 33%) | Not reported | High school: 14 (13.6%); Certificate level: 31 (30.1%); Undergrad: 46 (44.7%); Postgrad: 12 (11.7%) | See Supplementary 7 | Number of residents participants live with (M = 1.67, SD = 1.2) | M = 34.7; SD = 10.50 |
| Backman et al. (2024) | Not reported | Not reported | Independent employment or student (n = 9; 66.7%); supported employment (n = 21; 14,9%); not working/studying (n = 8; 5.7%) | Female (n = 92; 65.2%); male (n = 49; 34.8%) | Not reported | Upper secondary: 72 (54.1%); College/un: 5 (3.6%) | Not reported | Not reported | M = 20.46; SD = 2.96 |
| Bennett et al. (2021) | Not reported | Not reported | Not reported | Female (n = 17; 50%); male (n = 17; 50%) | Not reported | Not reported | Not reported | Not reported | M = 11.2; SD = 3.3 |
| Bilan et al. (2025) | Not reported | Not reported | Not reported | Female (n = 6; 15%); male (n = 35; 85%) | Not reported | Not reported | Not reported | Not reported | Intervention M = 9.41 (SD = 1.22); Control M = 9.38 (SD = 1.21) |
| Carl et al. (2020) | Not reported | Asian (n = 7; 3%); Black (n = 10; 4%); Multiple (n = 13; 5%); White (n = 215; 84%); Other (n = 1; 0%) | Not reported | Female (n = 175; 68%); male (n = 77; 30%); other (n = 4; 2%) | Not reported | No formal qualifications: Control: 2 (2%), Intervention: 3 (2%); Secondary school/high school graduate: Control: 20 (16%), Intervention: 19 (15%); College: Control: 31 (24%), Intervention:35 (27%); Undergraduate/bachelor's degree: Control: 50 (39%), Intervention: 42 (33%); Postgraduate or professional degree: Control: 25 (20%), Intervention: 29 (23%) | Not reported | Married/civil partnership (n = 68; 26%); separated (n = 4; 2%); divorced (n = 15; 6%); never married/single (n = 123; 43%); partnered (n = 44; 17%); prefer not to say (n = 2; 1%) | M = 30.9 years |
| Chan et al. (2023) | Not reported | Not reported | Not reported | Female (n = 22; 73%); male (n = 87; 27%) | Not reported | All participants completed high school, and 90% had attained some university education | Not reported | Married (n = 23; 7.2%) | M = 27.3; SD = 7.2 |
| Chien et al. (2024) | Not reported | Not reported | Not reported | Female (n = 47; 84%); male (n = 3; 6%) | Not reported | Associate degree or above: Intervention - n=9; control - n=14 | See Supplementary 7 | Married (n = 48; 96%) | Intervention M = 35.92 (SD = 5.99); Control M = 39.44 (SD = 3.72) |
| Clark et al. (2023) | Not reported | White (n = 90; 88%) | Employed (n = 78; 76%); not employed (n = 10; 10%) | Female (n = 53; 52%); male (n = 49; 48%) | Not reported | Primary 14%, Secondary 21%, further 65% | Not reported | Married or in long-term relationship (n = 55; 54%) | M = 32.2; SD = 8.3 |
| Creswell et al. (2024) | Not reported | White (n = 400; 90%); multiple (n = 33; 7.5%); Asian (n = 3; 0.7%); Black (n = 2; 0.5%); other (n = 2; 0.5%) | Employed (74%); not employed (7.22%) | Female (n = 255; 58%); male (n = 184; 42%); other (n = 3; 0.7%) | Not reported | Parent 1 data  School completion: OSI = 35 (16%), TAU = 33 (15%)  Further education: OSI = 103 (46%), TAU =101 (46%)  Higher education: OSI = 39 (18%), TAU =53 (24%)  Postgraduate qualification: OSI = 45 (20%), TAU = 34 (15%) | See Supplementary 7 | Partnered (n = 353; 80%) | Intervention M = 9.31 (SD = 1.83); Control M = 9.08 (SD = 1.74) |
| Dopfner et al. (2025) | Not reported | Not reported | Not reported | Female (n = 392; 91%); male (n = 39; 9%) | Not reported | Parent data: Primary and lower secondary level of education:  12.9%; Upper secondary level of education: 27.6%; Upper secondary level of education (ISCED  level 3A) and post-secondary non-tertiary:  19.0%; First stage of tertiary education: 12.9%; First stage (ISCED level 5A) or second stage of  tertiary education (ISCED level 6): 27.6% | Not reported | Not reported | Child age: M = 9.35 (SD = 1.68) |
| Eto et al. (2025) | Not reported | Not reported | Employed (n = 22; 73.3%); not employed (n = 8; 26.7%) | Female (n = 16; 53.3%); male (n = 14; 46.7%) | Not reported | Length of education: Intervention= 15.7 years ± 1.79, Control: 16.9 years ± 2.70 | Not reported | Married (n = 11; 36.7%) | Intervention M = 34.2 (SD = 9.61); Control M = 37.3 (SD = 11.38) |
| Eylem et al. (2021) | Not reported | Not reported | Employed (n = 5; 27.7%); not employed (n = 13; 72.2%) | Female (n = 13; 72.2%); male (n = 5; 27.8%) | Not reported | Secondary school: 4 (22.2%); University: 8 (44.4%); Other: 6 (33.3%) | Not reported | Single (n = 11; 61.1%); in relationship (n = 1; 5.5%); married (n = 4; 22.2%); widowed (n = 2; 11.1%) | M = 33.5; SD = 8.38 |
| Fatouros et al. (2025) | Not reported | Not reported | Not reported | Female (n = 154; 77%); male (n = 46; 23%) | Not reported | Not reported | Not reported | Not reported | M = 34.37; SD = 9.33 |
| Felder et al. (2020) | Not reported | White (n = 138; 66.3%); Hispanic (n = 15; 7.2%) | Not reported | Female (n = 208; 100%) | Not reported | Secondary school: 4 (22.2%); University: 8 (44.4%); Other: 6 (33.3%) | See Supplementary 7 | Married or cohabiting (n = 196; 94.2%) | Intervention M = 33.9 (SD = 3.38); Control M = 33.21 (SD = 3.98) |
| Felder et al. (2022) | Not reported | White (n = 138; 66.3%); Hispanic (n = 15; 7.2%) | Not reported | Female (n = 208; 100%) | Not reported | Secondary school: 4 (22.2%); University: 8 (44.4%); Other: 6 (33.3%) | See Supplementary 7 | Married or cohabiting (n = 196; 94.2%) | Intervention M = 33.9 (SD = 3.38); Control M = 33.21 (SD = 3.98) |
| Grenier-Martin et al. (2022) | Not reported | Not reported | Employed (n = 22; 75.8%); not employed (n = 7; 24.1%) | Female (n = 25; 86.2%); male (n = 4; 13.8%) | Not reported | Secondary school not completed: Intervention: 1 (6.3%), Control: 0 (0.0%); Vocational diploma: Intervention: 1 (6.3%), Control: 1 (7.7%); College diploma: 4 (25.0%), Control: 5 (38.5%); Bachelor’s degree: Intervention: 6 (37.5%), Control: 4 (30.8%); Master’s/PhD degree: Intervention: 4 (25.0%), Control: 3 (23.1%) | See Supplementary 7 | Not reported | Child age: M = 4 |
| Guzick et al. (2023) | All required to be full-time residents in Texas, USA | White (n = 5; 63%); multiple (n = 2; 25%); Black (n = 1; 13%) | Not reported | Female (n = 3; 38%); male (n = 4; 50%) | Not reported | Only mothers education used in the categorisation. Mother education: Associates degree/high school diploma: 1 (13%), Bachelors Degree: 4 (50%), Graduate Degree: 3 (38%) | See Supplementary 7 | Not reported | Child age: M = 10.3; SD = 3.3 |
| Guzick et al. (2024) | All required to be full-time residents in Texas, USA | White (n = 40; 70%); Asian (n = 8; 14%); Black (n = 3; 5%); multiple (n = 3; 5%); other (n = 3; 5%) | Not reported | Female (n = 16; 28%); male (n = 41; 72%) | Not reported | Parent 1 education  High school diploma: Total = 2 (4%),  Some college: Total = 6 (11%),  Technical/trade/vocational school: Total = 1 (2%),  Associate's degree: Total = 2 (4%),  Bachelor's degree: Total = 19 (33%),  Graduate degree: Total = 27 (47%), | Not reported | Not reported | Child age: M = 11.05; SD = 2.54 |
| Hall et al. (2024) | Not reported | N/A (perspective piece) | N/A | N/A | N/A | N/A | N/A | N/A | N/A |
| Hartley et al. (2022) | Not reported | Not reported | Not reported | Female (n = 13; 81.3%); male (n = 1; 6.3%); other (n = 2; 12.5%) | Not reported | Post-graduate degree: Adult=1, Caregiver= 3  Degree or diploma: Adult=1, Caregiver= 7  High school: Adult=1, Caregiver = 1  Primary school: 0 | Not reported | Not reported | M = 35.0 (SD = 10.0) |
| Haun et al. (2023) | Rural (n = 66; 18%); urban = 298; 81.9%) | White (n = 395; 74.5%); Black (n = 78; 14.7%); other (n = 24; 4.5%); multiple (n = 22; 4.2%); Asian (n = 11; 2.1%) | Not reported | Female (n = 285; 53.8%); male (n = 242; 45.7%); other (n = 3; 0.6%) | Not reported | veterans intervention: none (0%) = less than a high school education, 11 (8.3%)= completed high school, 34 (25.6%) = some college or vocational school, 31 (23.3%) = an associate degree, 32 (24.1%) = bachelor’s degree, 25 (18.8%) = graduate degree, 0 (0%) = declining to respond.  veteran control: none (0%) = less than a high school education, 6 (4.6%) = completed high school, 39 (29.6%) = some college or vocational school, 29 (22%) = an associate degree, 33 (25%) = a bachelor’s degree, 25 (18.9%) = a graduate degree, 0 (0%) = declining to respond.  partners intervention: none (0%) = less than a high school education, 18 (13.5%) = completed high school, 42 (31.6%) = some college or vocational school, 20 (15%) = an associate degree, 32 (24.1%) = a bachelor’s degree, 21 (15.8%) = a graduate degree, 0 (0%)= declining to respond.  partner control: 1 (0.8%) = less than a high school education, 23 (17.4%) = completed high school, 30 (22.7%) = some college or vocational school, 27 (20.5%) = an associate degree, 27 (20.5%) = a bachelor’s degree, and 23 (17.4%) = a graduate degree, 1 (0.8%) = declining to respond. | Not reported | Veterans married or partnered (n = 203; 76.6%); divorced, separated or widowed (n = 54; 20.4%); single or never married (n = 7; 2.6%) | Intervention M = 55.8 (SD = 14.1); Control M = 57.3 (SD = 13.4) |
| He et al. (2022) | Rural (n = 80; 54.1%); urban (n = 68; 45.9%) | Asian (n = 137; 92.5%) | Not reported | Female (n = 55; 37.2%); male (n = 93; 62.8%) | Not reported | University students | Not reported | Not reported | M = 18.78; SD = 0.88 |
| Heller et al. (2020) | Not reported | White (n = 134; 84%) | Employed (n = 111; 70%) | Female (n = 159; 100%) | Not reported | Low: Intervention: 4 (5%); Control: 0 (0%);  Middle: Intervention: 14 (18%); Control: 21 (26%); High: Intervention: 61 (77%); Control= 59 (74%) | Not reported | Not reported | Intervention M = 32.08 (SD = 4.61); Control M = 31.94 (SD = 4.83) |
| Hoffmann et al. (2021) | Not reported | Not reported | Employed (N = 73; 72%); not employed (n = 17; 17%) | Female (n = 66; 65%); male (n = 35 (35%) | Not reported | Unskilled: iact = 6 (11%), iforum= 4 (8%)  Skilled: iact = 5 (9%), iforum = 7 (15%)  Higher education (<4 years): Iact = 28 (53%), iforum = 22 (46%)  Higher education (>4 years): iact = 11 (21%), iforum = 14 (29%)  Other: iact = 3 (6%), iforum = 1 (2% | Not reported | Married or living with partner (n = 78; 77.2%) | M = 37.2; SD = 9.7 |
| Hollis et al. (2021) | Not reported | White (n = 195; 87%); Asian (n = 10; 4.5%); multiple (n = 10; 4.5%); Black (n = 1; 0.4%); other (n = 1; 0.4%) | Employed (n = 183; 82%); not employed (n = 40; 18%) | Female (n = 47; 21%); male (n = 177; 79%) | Not reported | Mother  no qual: CONT: 1 (1%), INT: 3 (3%)  Mandatory secondary education (eg, GCSEs): CONT: 17 (15%), INT: 16 (14%)  Further education (eg, A-levels, BTEC, NVQ): CONT: 32 (29%), INT: 33 (29%)  Higher education (eg, BA, BSc): CONT: 46 (41%), INT: 46 (41%)  Postgraduate education (eg, MA, MSc, PhD): CONT: 16 (14%), INT: 14 (13%) | Not reported | Not reported | Child age: Intervention M = 12.2 (SD = 2.0); Control M = 12.4 (SD = 2.1) |
| Huberty et al. (2021) | Not reported | White (n = 152; 59.2%); Black (n = 49; 20.4%); Asian (n = 28; 11.7%); other (n = 15; 6.3%) | Not reported | Female (n = 187; 77.9%); male (n = 53; 22.1%) | Not reported | Not reported | Not reported | Not reported | Intervention M = 44.15 (SD = 14.1); Control M = 44.24 (SD = 15.1) |
| Jamali et al. (2022) | Not reported | Not reported | Not reported | Females (n = 10; 23.3%); males (n = 33; 76.7%) | Not reported | Parental education: Primary: INT = 4, CONT = 2  Finished secondary: INT =5, CONT = 9  Associate or bachelor’s degree: INT =9, CONT = 11  Master’s degree: INT =3, CONT = 0 | Not reported | Not reported | Child age: Intervention M = 8.18 (SD = 2.32); Control M = 8.48 (SD = 2.84) |
| Jent et al. (2021) | Not reported | White (n = 130; 74.9%); multiple (n = 20; 11.5%); Black (n = 16; 9.2%); other (n = 5; 2.8%); Asian (n = 2; 1.15%) | Not reported | Male (n = 57; 31.15%) | Not reported | Caregiver data: < College degree: PCIT=23 (32.43%), PCIT+Ebook = 29 (27.36%);  ≥ 4-year college degree: PCIT=48 (67.57%), PCIT+Ebook=77 (72.64%) | Not reported | Not reported | Intervention M = 38.10 (SD = 6.45); Control M = 38.60 (SD = 10.66) |
| Kalmbach et al. (2020) | Not reported | White (n = 47; 51.6%); Black (n = 29; 31.9%); Asian (n = 6; 6.6%); other (n = 6; 6.6%); multiple (n = 3; 3.3%) | Not reported | Female (n = 91; 100%) | Not reported | Not reported | See Supplementary 7 | Not reported | M = 29.03; SD = 4.16 |
| Kandola et al. (2024) | Not reported | Not reported | Not reported | Female (n = 356; 78.07%); male (n = 72; 15.69%); non-binary (n = 30; 6.58%) | Not reported | Not reported | Not reported | Not reported | M = 33.41; SD = 12.35 |
| Kenworthy et al. (2023) | Not reported | White (N = 71; 73.96%); multiple (n = 12; 12.50%); Black (n = 8; 8.33%); Asian (n = 5; 5.21%) | Not reported | Female (n = 89; 92.71%) | Not reported | Parent education years, Mean (SD): In-person = 16.68 (2.03); online = 17.04 (2.28) | Not reported | Not reported | Child age: Intervention M = 9.8 (SD = 1.43); Control M = 9.89 (SD = 1.52) |
| Kwon et al. (2024) | Not reported | Not reported | Not reported | Female (n = 13; 17.6%); male (n = 61; 82.4%) | Not reported | Not reported | Not reported | Not reported | Child age M = 9.92; SD = 1.93 |
| Lewis et al. (2024) | Rural (n = 12; 19.6%); urban (n = 49; 80.4%) | Not reported | Not reported | Female (n = 28; 45.9%); male (n = 30; 49.2%); other (n = 3; 4.9%) | Not reported | Did not complete high school: Intervention = 8 (26.7%), TAU = 5 (16.1%); Completed high school only: Intervention = 1 (3.3%), TAU = 3 (9.7%); Completed tertiary study: Intervention = 29 (96.7%), Control = 28 (90.3%) | See Supplementary 7 | Single parent household (n = 9; 14.8%) | Intervention M = 42.56 (SD = 5.37); Control M = 44.45 (SD = 7.09) |
| Lindgren et al. (2020) | Not reported | Not reported | Not reported | Female (n = 6; 15.8%); male (n = 32; 84.2%) | Not reported | Not reported | Not reported | Not reported | Intervention M = 49.71 (SD = 16.97); Control M = 55.29 (SD = 17.01) |
| Lippke et al. (2021) | Not reported | Not reported | Employed (n = 227; 75.7%) | Female (n = 199; 66.3%) | Not reported | Education – Elementary school:  Superiority CAU = 18 (dropouts), 23 (completers)  Superiority ONL2 = 19 (dropouts), 22 (completers)  Equivalence F2F = 16 (dropouts), 26 (completers)  Equivalence ONL1 = 16 (dropouts), 11 (completers) Education – High school:  Superiority CAU 6 (dropouts), 10 (completers)  Superiority ONL2 = 14 (dropouts), 10 (completers)  Equivalence F2F = 2 (dropouts), 4 (completers)  Equivalence ONL1 = 5 (dropouts), 9 (completers)Education – College and above:  Superiority CAU = 9 (dropouts), 9 (completers)  Superiority ONL2 = 13 (dropouts), 10 (completers)  Equivalence F2F = 13 (dropouts), 9 (completers)  Equivalence ONL1 = 11 (dropouts), 10 (completers)Education – Other:  Superiority CAU = 2 (dropouts), 2 (completers)  Superiority ONL2 = 0 (dropouts), 0 (completers)  Equivalence F2F = 0 (dropouts), 0 (completers)  Equivalence ONL1 = 0 (dropouts), 1 (completers) | See Supplementary 7 | Married (n = 157; 52.3%) | M = 50.23; SD = 9.78 |
| Malarkey et al. (2024) | Not reported | White (n = 78; 73.6%); Black (n = 11; 10.4%); other (n = 7; 6.6%); Asian (n = 5; 4.7%); multiple (n = 5; 4.7%) | Employed (n = 84; 79.3%); not employed (n = 21; 19.8%) | Female (n = 23; 21.7%); male (n = 87; 78.3%) | Not reported | High school degree or less:   Control = 2 (8.3%), eCBT-I = 11 (13.4%)  Some college or college degree:   Control = 9 (37.5%), eCBT-I = 41 (50.0%)  Graduate degree:   Control = 13 (54.2%), eCBT-I = 30 (36.6%) | Not reported | Not reported | Intervention M = 42 (SD = 10); Control M = 42 (SD = 12) |
| March et al. (2023) | Rural (n = 11; 10%); urban (n = 99; 84.3%) | Not reported | Not reported | Female (n = 81; 59.12%); male (n = 56; 40.88%) | Not reported | Not reported | See Supplementary 7 | Not reported | Intervention M = 10.76 (SD = 2.40); Control M = 10.57 (SD = 2.46) |
| March et al. (2025) | Rural (n = 34; 25%); urban (n = 84; 61%) | Not reported | Not reported | Female (n = 76; 55%); male (n = 61; 45%) | Not reported | Not reported | See Supplementary 7d | Not reported | Intervention M = 11.08 (SD = 2.35); Control M = 10.79 (SD = 2.18) |
| McCloud et al. (2020) | Not reported | Not reported | Not reported | Female (n = 143; 85.1%); male (n = 23; 13.7%) | Not reported | University students studying in the UK | Not reported | Not reported | Intervention M = 25.1 (SD = 7.68); Control M = 23.5 (SD = 5.53) |
| McLellan et al. (2024) | Rural (n = 28; 29%) | Not reported | Not reported | Female (n = 91; 96%); male (n = 4; 4%) | Not reported | Primary Caregiver Highest Education:  University/ Doctoral degree: iCBT = 36 (76.6), wait =35 (72.9)  Vocational/ College: iCBT = 9 (19.1), wait =11 (22.9)  Primary/ High school: iCBT = 2 (4.3), wait = 2 (4.2) | See Supplementary 7 | Number of two-parent families (n = 86; 90.5%) | Child age: Intervention M = 9.26 (SD = 1.67); Control M = 9.02 (SD = 1.48) |
| Mechler et al. (2022) | Not reported | Not reported | Not reported | Female (n = 227; 84%); male (n = 36; 13%) | Not reported | Not reported | Not reported | Not reported | Intervention M = 17.29 (SD = 1.28); Control M = 17.35 (SD = 1.25) |
| Moshe et al. (2022) | Not reported | Not reported | Not reported | Female (n = 149; 58.9%); male (n = 104; 41.1%) | Not reported | Low = 171, Middle = 45, High = 37 | Not reported | Single (n = 34; 13.4%); in a relationship or married (n = 180; 71.1%); divorced or separated (n = 39; 15.4%) | M = 51.1; SD = 8.88 |
| Murray et al. (2021) | Not reported | Not reported | Not reported | Female (n = 213; 70.5%); male (n = 89; 29.5%) | Not reported | Not reported | Not reported | Not reported | Intervention M = 44.5 (SD = 10.9); Control M = 44.6 (SD = 12.4) |
| Nardi et al. (2022) | Not reported | Not reported | Employed (n = 19; 70.3%); not employed (n = 8; 29.6%) | Female (n = 25; 93%); male (n = 2; 7%) | Not reported | College or technical school: n = 7 (26%); 2-year degree: n = 2 (7%); 4-year degree: n = 5 (19%); master's degree: n = 12 (44%); PhD: n = 1 (4%) | See Supplementary 7 | Not reported | M = 42.9; SD = 15.6 |
| Nissling et al. (2023) | Not reported | Not reported | Employed (n = 4; 7.7%); not employed (n = 48; 92.3%) | Female (n = 43; 82.7%); male (n = 9; 17.3%) | Not reported | Early =13, Primary =34, Secondary =5 | Not reported | Living with someone from family (n = 47; 90.4%); lives with another adult (n = 1; 1.9%); lives alone (n = 2; 3.8%) | Intervention M = 16.63 (SD = 1.39); Control M = 16.6 (SD = 1.04) |
| Nordh et al. (2021) | Not reported | Not reported | Employed (n = 95; 92%); n = 8 (8%) | Female (n = 79; 77%); male (n = 24 (23%) | Not reported | <12y = 7 (7%), 12y = 7 (7%), Undergrad = 27 (26%), Graduate = 54 (52%) Postgrad = 8 (8%) | Not reported | Not reported | M = 14.1; SD = 2.1 |
| Ong et al. (2024) | Not reported | White (n = 120; 67.4%); other (n = 28; 15.7%); Black (n = 26; 14.6%) | Not reported | Female (n = 52; 29.2%); male (n = 125; 70.2%) | Not reported | Not reported | Not reported | Not reported | Intervention M = 47.66 (SD = 9.31); Control M = 48.9 (SD = 8.49) |
| Piscitello et al. (2024) | Not reported | White (n = 39; 90.7%); Black (n = 3; 7%); Asian (n = 2; 4.7%) | Not reported | Female (n = 34; 79.1%); male (n = 9; 20.9%) | Not reported | Secondary =1, Further =10, Higher =32 | See Supplementary 7 | Single (n = 8; 18.6%); married (n = 27; 62.8%); divorced or separated (n = 8; 18.6%) | M = 41.81; SD = 7.86 |
| Possemato et al. (2022) | Not reported | White (n = 60; 74%); Black (n = 8; 10%); multiple (n = 8; 10%); other (n = 5; 6%) | Employed (n = 25; 31%); not employed (n = 56; 69%) | Female (n = 33; 41%); male (n = 48; 59%) | Not reported | Secondary =11, Further = 49, Higher = 31 | Not reported | Single (n = 7; 9%); married or partnered (n = 36; 44%); separated or divorced (n = 30; 37%); widowed (n = 4; 5%) | M = 54; SD = 9.4 |
| Richards et al. (2020) | Not reported | White (n = 304; 84.2%); other (n = 57; 15.8%) | Employed (n = 268; 74.5%); not employed (n = 92; 25.5%) | Female (n = 258; 71.5%); male (n = 103; 28.5%) | Christian (n = 88; 24.4%); other (n = 52; 14.4%); none (n = 221; 61.2%) | Not reported | Not reported | Not reported | Median = 29; IQR = 18 |
| Sabri et al. (2025) | Not reported | Not reported | Not reported | Female (n = 144; 100%) | Not reported | Secondary =5, Further =18, Higher =103 | Not reported | In relationship (n = 86; 60%); not in a relationship (n = 50; 34.7%) | M = 33.6; SD = 8.05 |
| Sayal et al. (2025) | Not reported | White (n = 1022; 83%); Asian (n = 47; 4%); multiple (n = 21; 2%); Black (n = 15; 1%); other (n = 12; 1%) | Not reported | Female (n = 1044; 85.22%); male (n = 75; 6%) | Not reported | Not reported | See Supplementary 7 | Not reported | 5-10 years (n = 422; 34.4%); 11-15 years (n = 644; 52.6%); 16-17 years (n = 159; 13.0%) |
| Segal et al. (2020) | Not reported | White (n = 419; 91.9%); other (n = 26; 5.7%); Black (n = 8; 1.8%); Asian (n = 7; 1.5%) | Employed (n = 310; 67.4%); not employed (n = 149; 32.4%) | Female (n = 346; 75.6%); male (n = 122; 24.4%) | Not reported | Primary =6, Secondary =57, Further = 393 | See Supplementary 7 | Never married (n = 103; 22.5%); married or civil union (n = 217; 47.5%); divorced or separated (n = 120; 26.3%); widowed (n = 17; 3.7%) | M = 48.3; SD = 14.9 |
| Seo et al. (2022) | Not reported | Not reported | Employed (n = 22; 30.1%); not employed (n = 31; 69.9%) | Female (n = 73; 100%) | None (n = 38; 52.1%); Buddhist (n = 18; 24.7%); Christian (n = 13; 17.8%); Catholic (n = 4; 5.5%) | Secondary =3, Higher = 70 | See Supplementary 7 | Not reported | <30 (n = 10; 13.7%); 30-34 (n = 37; 50.7%); >35 (n = 26; 35.6%) |
| Sun et al. (2021) | Not reported | Asian (n = 168; 100%) | Not reported | Female (n = 168; 100%) | Not reported | Not reported | See Supplementary 7 | Married (n = 164; 97.6%) | M = 29.91; SD = 4.015 |
| Tan et al. (2023) | Not reported | Asian (n = 47; 97.9%); other (n = 1; 2.1%) | Employed (n = 28; 58.3%); not employed (n = 20; 41.7%) | Female (n = 37; 77.1%); male (n = 11; 22.9%) | Muslim (n = 38; 79.2%); other (n = 10; 20.8%) | No tertiary education = 15 (31.25%); Tertiary education = 33 (68.8%) | Not reported | Single (n = 31; 64.6%); married (n = 16; 33.3%); divorced (n = 1; 2.1%) | Intervention M = 26 (IQR = 24-30.75); Control M = 27 (IQR = 24.25-35.75) |
| Tan et al. (2024) | Not reported | Not reported | Not reported | Female (n = 67; 97.1%); male (n = 2; 2.9%) | Not reported | Secondary = 2, Further =11, Higher =56 | See Supplementary 7 | Married (n = 65; 94.2%); divorced, separated or widowed (n = 4; 5.8%) | M = 40.72; SD = 4.56 |
| Wong et al. (2021) | Not reported | Not reported | Employed (n = 31; 39.2%); not employed (n = 48; 60.7%) | Female (n = 67; 84.8%); male (n = 12; 15.2%) | Not reported | Secondary =1, Further = 5, Higher =73 | See Supplementary 7 | Single (n = 59; 74.7%); married (n = 18; 22.8%); divorced or widowed (n = 2; 2.6%) | M = 32.9; SD = 12.5 |
| Wu et al. (2023) | Not reported | Not reported | Not reported | Female (n = 37; 39.8%); male (n = 56; 60.2%) | Not reported | years of education with a mean of 13.82 years (SD = 2.79) in in-person CBT group; 13.81 (SD = 3.31) in internet-based CBT group, and 14.55 (SD = 2.41) in TAU group. | Not reported | Married (n = 56; 60.2%); not married (n = 37; 39.8%) | Intervention M = 29.16 (SD = 6.35); Control M = 30.29 (SD = 8.09) |
| M = mean; SD = standard deviation; IQR = interquartile range | | | | | | | | | |

**S4b Table. Sources used to determine sociodemographic characteristics of the country of study***

| **Sociodemographic Characteristic** | **Country** | **Source** |
| --- | --- | --- |
| Place | Australia | <https://www.abs.gov.au/statistics/people/population> |
| Race | USA | https://www.census.gov/library/stories/2021/08/improved-race-ethnicity-measures-reveal-united-states-population-much-more-multiracial.html |
|  | UK | <https://www.ethnicity-facts-figures.service.gov.uk/uk-population-by-ethnicity/national-and-regional-populations/population-of-england-and-wales/latest/#:~:text=according%20to%20the%202021%20Census,up%20from%20333%2C100%20to%20923%2C800> |
| Occupation | USA | <https://www.census.gov/programs-surveys/decennial-census/decade/2020/2020-census-results.html> |
|  | UK | <https://www.ons.gov.uk/employmentandlabourmarket/peopleinwork/employmentandemployeetypes/bulletins/employmentintheuk/february2025> |
|  | Sweden | <https://unece.org/sites/default/files/2024-04/BAS_QoE.pdf#:~:text=With%20a%20population%20of%207%2C6%20(10%2C5)%20million,out%20of%20them%2089.7%20percent%20were%20employees> |
| Gender/Sex | USA | <https://www.census.gov/programs-surveys/decennial-census/decade/2020/2020-census-results.html> |
|  | UK | <https://www.ons.gov.uk/aboutus/transparencyandgovernance/freedomofinformationfoi/populationoftheukbysex> |
|  | Australia | <https://www.abs.gov.au/statistics/people/population> |
|  | Sweden | <https://www.scb.se/en/finding-statistics/statistics-by-subject-area/population-and-living-conditions/population-composition-and-development/population-statistics/pong/tables-and-graphs/population-statistics---summary/swedens-population-in-summary-1960-2024/> |
|  | China | <https://www.stats.gov.cn/english/PressRelease/202105/t20210510_1817189.html#:~:text=Of%20the%20national%20population%5B2,National%20Population%20Census%20in%202010> |
|  | Germany | <https://tradingeconomics.com/germany/population-female-percent-of-total-wb-data.html#:~:text=Population%2C%20female%20(%25%20of%20total%20population)%20in,development%20indicators%2C%20compiled%20from%20officially%20recognized%20sources> |
|  | Hong Kong | <https://statisticstimes.com/demographics/country/hong-kong-demographics.php#:~:text=There%20are%203.34%20million%20males,male%20ratio%20in%20the%20world> |
|  | Denmark | <https://statisticstimes.com/demographics/country/denmark-demographics.php> |
|  | Spain | <https://statisticstimes.com/demographics/country/spain-demographics.php#:~:text=There%20are%2023.52%20million%20males,female%2Dto%2Dmale%20ratio> |
| Education | USA | <https://www.census.gov/programs-surveys/decennial-census/decade/2020/2020-census-results.html> |
|  | UK | <https://www.ons.gov.uk/peoplepopulationandcommunity/educationandchildcare/bulletins/educationenglandandwales/census2021#:~:text=For%20equivalent%20qualifications%2C%20see%20Measuring,18.2%25%2C%208.8%20million>) |
|  | Australia | <https://assets.publishing.service.gov.uk/media/5f6cbefed3bf7f72361878af/Appendices_International_comparisons_report_final_for_publication.pdf> |
|  | Germany | <https://gpseducation.oecd.org/Content/EAGCountryNotes/EAG2023_CN_DEU_pdf.pdf> |
|  | Hong Kong | <https://pmc.ncbi.nlm.nih.gov/articles/PMC9669390/> |
|  | Sweden | <https://www.statista.com/statistics/532459/sweden-population-2015-by-level-of-education/> |
|  | China | <http://en.moe.gov.cn/news/press_releases/202304/t20230403_1054080.html#:~:text=Higher%20education%20further%20expanded%20its,of%20all%20students%20in%20China> |
| Social Capital (Marriage status) | USA | <https://www.ons.gov.uk/peoplepopulationandcommunity/populationandmigration/populationestimates/bulletins/populationestimatesbymaritalstatusandlivingarrangements/2022> |
|  | China | Only possible to identify number of marriages registered over a set period |
|  | Hong Kong | Only possible to identify number of marriages registered over a set period |
|  | UK | <https://www.ons.gov.uk/peoplepopulationandcommunity/birthsdeathsandmarriages/marriagecohabitationandcivilpartnerships/articles/marriageandcivilpartnershipstatusenglandandwalescensus2021/2023-02-22> |
|  | Germany | <https://www.destatis.de/EN/Themes/Society-Environment/Population/Current-Population/Tables/population-by-marital-status.html> |

*SES/Income categorisations are reported in Supplementary Material 7
