# Supplementary material for: Who gets included? Equity in digital and decentralised mental health and neurodevelopmental trials: A systematic review
Source: PLOS Digit Health. 2026 Jun 8;5(6):e0001466. doi: 10.1371/journal.pdig.0001466 (PMC13245764; doi:10.1371/journal.pdig.0001466)
Supplement: S5 Table — S5a Table. References used for calculating high, medium and low income earners by country. S5b Table. References used for determining studies included in the poverty comparisons. (DOCX) [file pdig.0001466.s007.docx]

**S5 Table. Categorisations and data sources for socio-economic status (SES)/ income**

**S5a Table. References used for calculating high, medium and low income earners by country.**

| **Reference (paper)** | **Country** | **Data Reported** | **Reference for categorisation** |
| --- | --- | --- | --- |
| **March 2025** | Australia | Socio-Economic Index for Areas:  0-25th: self-guided = 6 (9%), therapist-guided =9 (13%)  26th-50th: self-guided =11 (17%), therapist-guided =14 (20)  51st-75th: self-guided =19 (29%), therapist-guided = 16 (23%)  76th-100th: self-guided = 27 (41%), therapist-guided =30 (42%)  Missing: self-guided = 3 (5%), therapist-guided =2 (3%) | <https://www.abs.gov.au/articles/new-census-insights-income-australia-using-administrative-data>  For income data ‘Middle’ weekly income was taken as $1770 (household) $789 (personal).  ‘Middle’ annual income was taken as $92040 (household) $ 41028 (personal) |
| **McLellan 2024** | Australia | Annual Household Income AUD $:  < 83,000: iCBT = 5 (10.6), wait=9 (18.8%) 84,000 – 167, 000: iCBT = 27 (57.4%), wait= 24 (50%)  168,000 – 268,000: iCBT = 10 (21.3%), wait=12 (25%)  269,000+: iCBT = 5 (10.7%), wait=3 (6.3%) | As above |
| **Andrews 2023** | Australia | Annual Income AUS $: 0-19,999 = 15 (14.6), 20-80,000 = 48 (46.6%), >80,000 = 29 (28.2%), plus 'not disclosed' = 11 (10.7%) | As above |
| **Lewis 2024** | Australia | Weekly income AUS $: less than $999:  Intervention = 4 (13.3%), TAU = 5 (16.1%)  $1000–$1799: Intervention = 3 (10.0%), TAU = 8 (25.8%); $1800–$2799: Intervention = 13 (43.3%), TAU = 9 (29.0%); $2800–$5499: Intervention = 7 (23.3%), TAU = 5 (16.1%); $5500 or more:  Intervention = 3 (10.0%), TAU = 3 (9.7%) | As above |
| **March 2023** | Australia | Socio-economic Index for Areas ≤ 25th percentile: SC = 6 (9.80%), TG = 6 (10.00%); 26th–50th percentile: SC = 6 (9.80%), TG = 14 (23.30%); 51st–75th percentile: SC =24 (39.30%), TG =21 (35.00%); ≥ 76th percentile: SC = 25 (41.00%), TG = 19 (31.70%) | As above |
| **Sun 2021** | China | Monthly Income US $: <309: n = 1 (0.7%); 309-618: n = 32 (21.1%); 618-926: n = 46 (30.3%); >926: n = 73 (48%) | https://chinapower.csis.org/china-middle-class/  Middle annual income was based on McKinsey figures: $11,500-$43,000, RMB: 75000-28000 |
| **Tan 2024** | China | Monthly family income RMB: Less than 4000: 1 (1.45%); 4,001-6,000: 4 (5.8%); 6,001-8,000: 6 (8.70%); 8,001-10,000: 9 (13.04%); 10,001-20,000: 19 (27.54%); more than 20,000: 30 (43.48%) | As above |
| **Chien 2024** | Hong Kong | Monthly family income equal to or greater than the median: intervention: 13 (52%), control: 14 (56%) | https://www.censtatd.gov.hk/en/web_table.html?id=130-06606  For income data, ‘Middle’ income was classified as 30,000HK$ |
| **Wong 2021** | Hong Kong | <HK$ 5,000: 39 (49.4%); HK$ 5,001-10,000 :6 (7.6%); HK$ 10,001-20,000 11 (13.9%); HK$20,001: 30,000 = 9 (11.4%), HK$30,001-50,000 = 8 (101%), and HK$50,001-70,000 = 6 (7.6%) | As above |
| **Creswell 2024** | UK | Total household income £:  ≤16 000 per year: OSI = 17 (12%), TAU =18 (13%)  16 001–30 000 per year: OSI = 27 (19%). TAU =25 (18%)  30 001–40 000 per year: OSI = 14 (10%), TAU =18 (13%)  40 001–50 000 per year: OSI = 11 (8%). TAU =12 (9%)  50 001–60 000 per year: OSI = 12 (9%), TAU =17 (13%)  60 001–70 000 per year: OSI = 11 (8%), TAU =7 (5%)  70 001–80 000 per year: OSI = 8 (6%), TAU =10 (7%)  80 001–90 000 per year: OSI = 6 (4%), TAU =5 (4%)  90 001–120 000 per year: OSI = 8 (6%), TAU =4 (3%)  >120 000 per year: OSI = 3 (2%), TAU =6 (4%)  Prefer not to say: OSI = 24 (17%), TAU =14 (10%) | [https://cy.ons.gov.uk/](https://cy.ons.gov.uk/peoplepopulationandcommunity/personalandhouseholdfinances/incomeandwealth/bulletins/householddisposableincomeandinequality/financialyearending2024#:~:text=2.,per%20year%20for%20this%20period.)  High: £50k Plus, Middle: £36-50k; Low: Below 30k |
| **Sayal 2025** | UK | Index of Multiple Deprivation quintile (child's primary residence)  1st quintile (most deprived): intervention = 112 (18%), control = 102 (17%)  2nd quintile: intervention =116 (19%), control = 122 (20%)  3rd quintile: intervention =143 (23%), control = 132 (22%)  4th quintile: intervention =105 (17%), control = 104 (17%)  5th quintile (least deprived): intervention =138 (22%), control = 149 (24%)  Missing: intervention =1, control = 1 | As above |
| **Felder 2020** | USA | Treatment as Usual group: 70 participants had income ≥ $100,000; Digital CBT-I group: 71 participants had income ≥ $100,000. | https://www.pewresearch.org/short-reads/2024/09/16/are-you-in-the-american-middle-class/  High = >$169800, Middle = $56001-169800, Low = $<56,600 |
| **Felder 2022** | USA | Treatment as Usual group: 70 participants had income ≥ $100,000; Digital CBT-I group: 71 participants had income ≥ $100,000 | As above |
| **Kalmbach 2020** | USA | Poverty: n= 16/90 (17.8%) | Classified as low |
| **Nardi 2022** | USA | Annual income $: 20,000-29,000: n = 2 (7%); 30,000-39,000: n = 3 (11%); 40,000 - 49,000: (n = 3 (11%); 50,000-59,000: n = 1 (4%); 70,000-79,000: n = 2 ( 7%); 80,000-89,000: n = 2 (7%); 100,000 - 149,000: n = 8 (30%); > 150,000: n = 4 (15%) | https://www.pewresearch.org/short-reads/2024/09/16/are-you-in-the-american-middle-class/ |
| **Piscitello 2024** | USA | Household annual income $: <25,000: n = 2 (4.7%); 25,000-50,000: n = 12 (27.9%); 50,000-75,0000: n = 11, (25.6%); 75,000-100,000: n = 7 (16.3%); 100,000 - 125,000: n = 3 (7%); 125,000-150,000: n = 1 ( 2.3%); >150,000: n = 7, (16.3%) | As above |
| **Segal 2020** | USA | Annual income $: 0-29,999: n = 49 (10.8%); 30,000-69,999: n = 184, (40.4%); 70,000-99,999: n = 109 (24.0%); over 100,000: n = 113 (24.8%) | As above |
| **Grenier-Martin 2022** | Canada | ≤$19,999: Intervention: 2 (12.5%), Control: 1 (7.7%); $20,000–39,999: Intervention: 1 (6.3%), Control: 2 (15.4%); $40,000–59,999: Intervention: 3 (18.8%), Control: 2 (15.4%); $60,000–79,999: Intervention: 1 (6.3%), Control: 1 (7.7%); $80,000–99,999: Intervention: 2 (12.5%), Control: 2 (15.4%); ≥$100,000: Intervention: 7 (43.8%), Control: 5 (38.5%) | <https://www.springfinancial.ca/blog/lifestyle/middle-class-income-in-canada-by-province>  Low:<$57,375 ($40-59,999)  Middle: $57,375 - $114,750 ($60,000-9999)  High: > $114,750 (>$100,000) |
| **Seo 2022** | South Korea | <1800: n = 2 (2.7%); 1800-2699: n = 24 (32.9%); 2700-3599: n = 19 (26.02%); 3600-4499: n = 15 (20.5%); >4500: 13 (17.8%) - Presumed monthly, measured in USD. | [https://early.app/average-salary/south-korea](https://early.app/average-salary/south-korea/#:~:text=As%20mentioned%20above%2C%20South%20Korea,3.815%20KRW%20(2%2C858.27%20USD).)  Low: <$2000 (<1800)  Middle: $2,000-3000 (1800-2699 and 2700-3599)  High: > $3000 (3600-4499 and >4500) |
| **Lippke 2021** | Germany | Income – < €1500:  Superiority CAU = 8 (dropouts), 11 (completers)  Superiority ONL2 = 13 (dropouts), 15 (completers)  Equivalence F2F = 11 (dropouts), 13 (completers)  Equivalence ONL1 = 9 (dropouts), 9 (completers) Income – €1500–3000:  Superiority CAU = 17 (dropouts), 16 (completers)  Superiority ONL2 = 23 (dropouts), 17 (completers)  Equivalence F2F = 9 (dropouts), 17 (completers)  Equivalence ONL1 = 15 (dropouts). 17 (completers) Income – > €3000:  Superiority CAU = 10 (dropouts), 17 (completers)  Superiority ONL2 = 10 (dropouts), 10 (completers)  Equivalence F2F = 11 (dropouts), 9 (completers)  Equivalence ONL1 = 8 (dropouts), 5 (completers) | <https://www.destatis.de/EN/Themes/Labour/Earnings/Branch-Occupation/_node.html>  Average $4634 – classified as they are - $1500 – low, $1500-3000 – middle, >$3000 high |

**S5b Table. References used for determining studies included in the poverty comparisons**

| **Country** | **% from Country in Poverty** | **Poverty Line Source** | **Author** | **Total n** | **Relevant poverty metric reported in the paper** | **n (%) in poverty metric in paper** |
| --- | --- | --- | --- | --- | --- | --- |
| **Australia** | 13.4% | <https://povertyandinequality.acoss.org.au/poverty/> |  |  |  |  |
|  |  | SES quintile | March 2025 | 137 | SES <25% (good representation) | 15 (10.9%) |
|  |  | $53404 - Annual family | McLellan 2024 | 95 | Annual household <$83k (poor representation) | 14 (14.7%) |
|  |  | $25428 - Annual single | Andrews 2023 | 103 | Annual income <$20k (good representation) | 15 (14.6%) |
|  |  | SES quintile | Lewis 2024 | 61 | Weekly income <$999  (poor representation) | 9 (14.7%) |
|  |  | $489 - Weekly single | March 2023 | 137 | SES<25% (good representation) | 12 (8.7%) |
| **China** | 0.04% (Gov statistics questioned) | https://insights.grcglobalgroup.com/poverty-alleviation-comparison-china-and-the-u-s-by-joshua-xu/ |  |  |  |  |
|  |  | $390 USD | Sun 2021 | 168 | Monthly in USD <309 (poor representation) | 1 (0.5%) |
|  |  | 2800 RMB/year | Tan 2024 | 69 | Less than 4k RMB/month (poor representation) | 1 (1.4%) |
| **Hong Kong** | 20.2% | https://www.oxfam.org.hk/en/news-and-publication/the-wealth-gap-in-hong-kong-surges-to-819-times-elderly-poverty-exceed-580000 |  |  |  |  |
|  |  |  | Chien 2024 | 50 | N/A -  Monthly family income equal to or greater than the median: | NA |
|  |  | HK$4,400 month - single income | Wong 2021 | 79 | HK$<5K month (good representation) | 39 (49.3%) |
| **UK** | 15% | <https://researchbriefings.files.parliament.uk/documents/SN07096/SN07096.pdf> |  |  |  |  |
|  |  | greater than or equal to £13k/year | Creswell 2024 | 443 | less or equal to £16k/year (acceptable representation) | 35 (7.9%) |
|  |  | 1st Quintile IMD | Sayal 2025 | 1225 | 1st Quintile IMD (good representation) | 214 (17.4%) |
| **USA** | 11.4% | <https://www.census.gov/library/publications/2024/demo/p60-283.html> |  |  |  |  |
|  |  |  | Felder 2020 | 208 | income ≥ $100,000 (can’t categorise) | NA |
|  |  |  | Kalmbach 2020 | 91 | Reported as living in poverty (good representation) | 16 (17.5%) |
|  |  |  | Nardi 2022 | 27 | Reporting categories started over the $15225 limit | NA |
|  |  |  | Piscitello 2024 | 43 | <$25k (poor representation) | 2 (4.6%) |
|  |  |  | Segal 2020 | 460 | <29,999k (poor representation) | 49 (10.6%) |
